# Resilience and receptivity worked in tandem to sustain a geothermal mat community amidst erratic environmental conditions

**DOI:** 10.1038/srep12179

**Published:** 2015-07-17

**Authors:** Wriddhiman Ghosh, Chayan Roy, Rimi Roy, Pravin Nilawe, Ambarish Mukherjee, Prabir Kumar Haldar, Neeraj Kumar Chauhan, Sabyasachi Bhattacharya, Atima Agarwal, Ashish George, Prosenjit Pyne, Subhrangshu Mandal, Moidu Jameela Rameez, Goutam Bala

**Affiliations:** 1 Department of Microbiology, Bose Institute, P-1/12 CIT Scheme VIIM, Kolkata, 700054, India; 2 Thermo Fisher Scientific, 403/404 B-Wing, Delphi, Hiranandani Business Park, Powai, Mumbai 400076 India; 3 Department of Botany, The University of Burdwan, Burdwan, West Bengal, PIN - 713104, India; 4 Thermo Fisher Scientific, 372 Udyog Vihar Phase II, Gurgaon, Haryana, 122016, India

## Abstract

To elucidate how geothermal irregularities affect the sustainability of high-temperature microbiomes we studied the synecological dynamics of a geothermal microbial mat community (GMMC) vis-à-vis fluctuations in its environment. Spatiotemporally-discrete editions of a photosynthetic GMMC colonizing the travertine mound of a circum-neutral hot spring cluster served as the model-system. In 2010 a strong geyser atop the mound discharged mineral-rich hot water, which nourished a GMMC continuum from the proximal channels (PC) upto the slope environment (SE) along the mound’s western face. In 2011 that geyser extinguished and consequently the erstwhile mats disappeared. Nevertheless, two relatively-weaker vents erupted in the southern slope and their mineral-poor outflow supported a small GMMC patch in the SE. Comparative metagenomics showed that this mat was a relic of the 2010 community, conserved via population dispersal from erstwhile PC as well as SE niches. Subsequently in 2012, as hydrothermal activity augmented in the southern slope, ecological niches widened and the physiologically-heterogeneous components of the 2011 “seed-community” split into PC and SE meta-communities, thereby reclaiming either end of the thermal gradient. Resilience of incumbent populations, and the community’s receptiveness towards immigrants, were the key qualities that ensured the GMMC’s sustenance amidst habitat degradation and dispersal to discrete environments.

Despite the wide acceptance of geothermal environments as abodes of ancient lifeforms and facilitators of primordial metabolisms^1^,^2^,^3^,^4^ it is still unclear how ecological constraints affect the stability and sustainability of high temperature microbiomes. We do not have a precise understanding of how the structures and functions of geothermal microbial communities respond to fluctuating physicochemical conditions of their hydrothermal habitats. Synecological investigation of geothermal communities over time and space becomes more important in view of the ephemeral nature of the geochemistry of hydrothermal systems, which puts a subtle question mark on the feasibility of the hydrothermal origin of life. This was first highlighted when return expeditions to the famous Rose Garden vent field in the East Pacific rise (the first deep-sea hydrothermal vent to be discovered) revealed its total deactivation and consequent annihilation of the associated biodiversity within a decade^5^,^6^. Interestingly, during the same expedition, a young vent field in the Rose Garden neighborhood, christened as the Rosebud, was found to support another nascent geothermal community^7^. Yet, the issue of sustainability of microbial communities thriving on a nutrient source as uncertain as geothermal discharge was never quite adequately addressed with specific geomicrobiological investigations.

In this study we used high-throughput metagenomic techniques to trace the structures and metabolic potentials of a spatiotemporally-erratic geothermal microbial mat community (GMMC) over three consecutive years and correlated the community dynamics data with the physicochemical fluctuations in the environment. In the process we identified those community attributes, and niche constraints and opportunities, which collectively helped the GMMC sustain in the face of habitat instabilities. Many aspects of the community dynamics of the present GMMC could find parallels in other microbial communities enduring environmental whimsicalities elsewhere, thereby explaining how microbial populations within community frameworks cope with uncertain habitat conditions in general.

## Results and Discussion

### Site description and sample identity

The Dhauliganga valley in the Garhwal Himalayas (in the state of Uttarakhand, India) encompasses several circum-neutral hydrothermal vents, which discharge up to 95 °C hot waters having low chlorine and silica content^8^. The hot spring cluster of our interest, christened as the “Pilgrim Terrace” (PT) after the holy shrines of the nearby areas, is located at Tapovan (30.492542 N / 79.631002 E), 15 km south-east of the Joshimath town on the Malari-Joshimath Road. This site was explored in the November months of 2010, 2011 and 2012, and data gathered thereof constituted the basis of this report. Topographically, PT is a massive mound of hydrothermally deposited travertine interspersed with iron, nickel, aluminium, silver and sulfur. Spatiotemporally unstable vents seated within this mound discharge hot waters with fluctuating vigor and mineral content.

**Figure 1:**
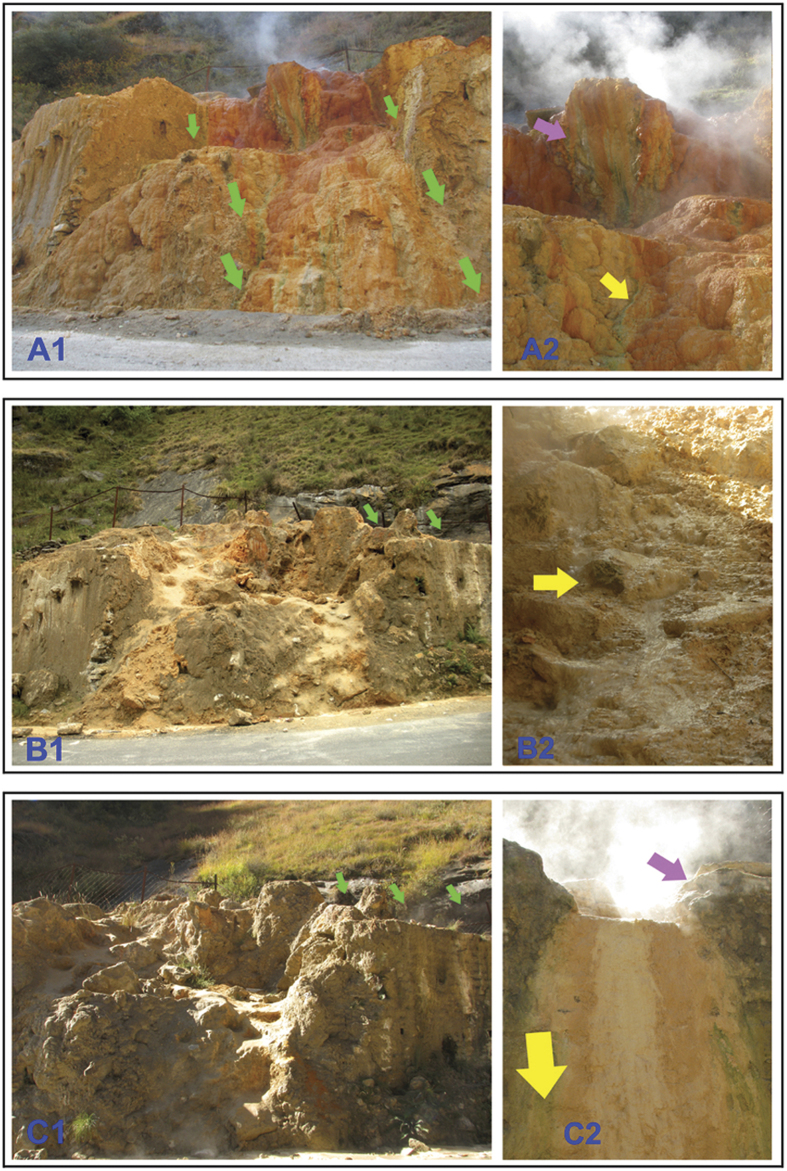
Changing microenvironments and associated microbial mats at the PT hot spring cluster between 2010 and 2012. **A1,B1 and C1** depicts the topography of the travertine mound and the position of the outflow channels (marked by green arrows) in 2010, 2011 and 2012 respectively. **A2, B2 and C2** shows the position of the green mats sampled in 2010, 2011 and 2012 respectively. Magenta arrows point towards the GM_PC samples while yellow arrows point at the GM_SE samples. **A1** depicts the vigorous hydrothermal discharge and heavy fumarolic activity of the large vent atop the Terrace in 2010, while **B1** and **C1** illustrate the cessation of this vent during the next two surveys. **A1** also shows that thermal outflows accompanied by active deposition of travertine and growth of green mats occurred along large tracts of the west-facing slope in 2010. **B1** and **C1**, on the other hand, illustrate the subsequent degradation of the west-slope microenvironment in 2011 and 2012 together with the concomitant shift of hydrothermal activities and microbial mats to the south-facing slope of the mound (topography of the southern slope not in view from the western side). All six photographs were taken by W. G.

**Figure 2:**
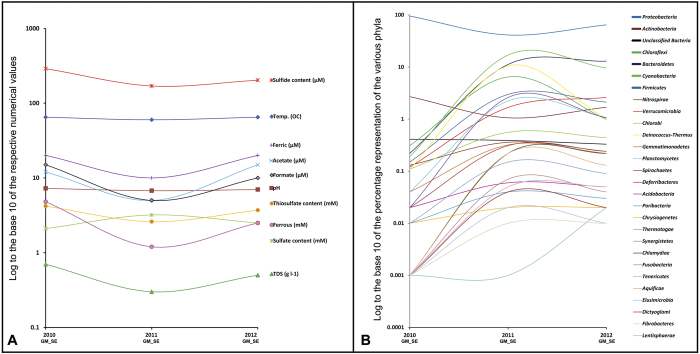
Fluctuation of physicochemical conditions in the SE (A) and relative abundance of bacterial phyla in the GM_SE community (B) over the three assessment years. Percentage of total classifiable metagenomic reads ascribed to a particular phylum represented the relative abundance of that phylum. In order to comprehensively compare highly disparate numbers log to the base 10 of all numerical values in question were plotted along the Y-axis of both **A** and **B**. Raw data used to generate ****A**** and **B** can be found in Table 1 and Table S2 respectively.

In 2010, vigorous hydrothermal discharge and fumarolic activity occurred from a large geyser situated on the flat top of the PT (Fig. 1A1). Thermal water (95 °C, pH 7.5) ejecting out of this vent (at a flow rate of ~20 l s^−1^) was rich in thiosulfate, sulfate and ferrous iron (Table 1, Fig. 2A). Active deposition of vertically-laminated red and creamy white sediments (Figure S1A) could be observed around the geyser mouth. Downslope, the laminated pattern gave way to fragile bulbous reddish yellow spherulites that entrapped fumarolic and/or microbially-produced gases (Figure S1B). Hyaline green microbial mats (GMs) grew all along the proximal as well as distal outflow channels (OFC) on the west-facing slope of the PT (area demarcated by green arrows in Fig. 1A1). GMs were sampled from the proximal channels (PC) as 2010_GM_PC, and also from the OFC in the slope environment (SE) as 2010_GM_SE (Fig. 1A2).

During our survey in 2011 we found that there was zero discharge from the PT-top geyser as its crater was completely clogged by sinter deposition. Consequently, there was no visible sign of the erstwhile GMMC along the west-facing slope of the PT (Fig. 1B1). Large chunks of the west-slope travertine had already been quarried, exposing hard old sinters from the deeper layers. The active sedimentary fabrics observed in the 2010 PCs were found vividly preserved in the texture of the old sinter layers exposed by fresh vertical cuts (Figure S1C). Elsewhere, on the eroded slopes of the remnant sinter, algae and mosses had started colonizing. Notwithstanding this scenario on the west-face, two relatively-weaker mid-terrace hot water vents were found to have formed anew on the south-facing slope of the PT where there was neither any hydrothermal feature nor any microbial mat in the previous year. Both had flow rates of ~4 l s^−1^, temperatures ~85 °C and pH 7.0. Their combined mineral-poor outflow (Table 1, Fig. 2A) drained down the southern slope and supported a small patch of deep green mat (sampled as 2011_GM_SE) on the thin layer of freshly-precipitated travertine (Fig. 1B2). Noticeably, this was the only visible counterpart of the sprawling 2010 GMMC in the entire PT microbiome of 2011.

Until 2012, there was no revival of the PT-top geyser (Fig. 1C1) and the western slope had started hosting angiospermous herbs (Figure S1D). However, hydrothermal manifestations in the southern slope had gained momentum as the two vents of 2011 origin had become more vigorous (flow rates ~6 l s^1^, 85–90 °C, pH 7.0–7.25), and just beneath the dead cavern of the 2010 geyser a small mud pool (~85 °C; pH 8.0) and a very strong but small vent (flow rate ~8 l s^−1^, ~95 °C, pH 7.5) formed anew. Discharge from this new vent joined the combined OFC of the 2011 vents below; and together the three created an outflow more vigorous and mineral-rich than last year. A small patch of deep green mat (sampled as 2012_GM_PC) grew on the PC close to the vents of 2011-origin (Fig. 1C2), while a couple of discrete GM stretches (sample 2012_GM_SE) also grew along the OFC in the SE.

### Rationale of the study and work-plan

For all practical purposes, our central objective was to know whether the small GM_SE patch of 2011 was a remnant of the expansive 2010 GMMC, and then whether the two niche-partitioned mats (GM_PC and GM_SE) of 2012 were derivatives of that 2011 GM_SE propagule. If these conjectures were found to be incorrect then it was imperative to confirm whether the 2011 and/or 2012 mats were distinct pioneer communities trying to colonize the PT afresh. It is further noteworthy that even if there was a spatiotemporal dispersal of the original west-slope community to the southern slope, its structure and function(s) were most likely to undergo alterations owing to the differences in the physicochemical conditions of the two sites. As such, the two types of changes needed to be identified and their potential correlations investigated.

Now, over the three assessment years five mat samples were retrieved from two distinct ecological niches, viz. the PC and SE OFCs. Incidentally, the SE niche was consistently occupied by mats throughout the study period but the PC niche was vacant in-between. Accordingly, the three GM_SE editions were chosen as the central models of this investigation and studied by both (i) shotgun metagenome analysis and (ii) amplified 16 S rDNA V3 sequence analysis. The two GM_PCs, on the other hand, were investigated mainly for comparative purposes, and V3 sequence-based taxonomic diversity analysis was sufficient for the same.

### Bacterial predominance in the GM_SE

**Figure 3:**
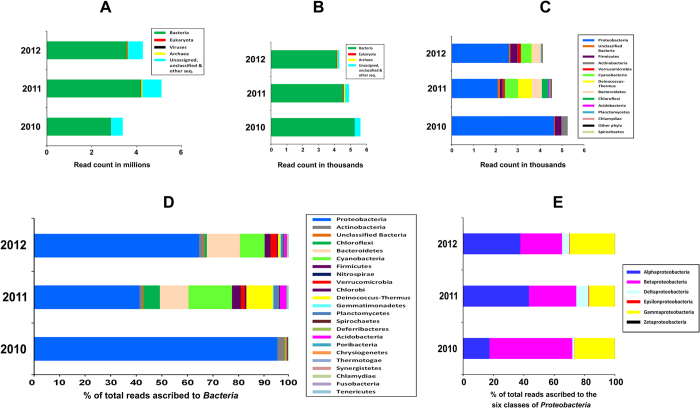
Shotgun metagenome analysis of the three GM_SE editions. **A**, Volume of data generated for each sample, and affiliation of total reads to the three domains, as found by searching (blastx) against the *nr* protein database using the “Organism Abundance” tool of the MG-RAST server following the “Best Hit Classification” (BHC) approach with minimum alignment length of 45 bp (15 amino acids) and minimum identity cutoff of 60%. **B**, Volume of small subunit (*ssu*) rRNA gene (rDNA) sequence encompassed by each dataset, and their distribution over the three domains, as found by searching (blastn) against the RDP database using the “Organism Abundance” tool of MG-RAST following the BHC approach with minimum alignment length of 50 bp and 70% minimum identity cutoff. **C**, Distribution of the bacterial *ssu* rDNA reads over various phyla. Other phyla included no read for 2010_GM_SE; a total of only 35 reads for 2011_GM_SE distributed over *Fusobacteria*, *Tenericutes*, *Aquificae*, *Fibrobacteres*, *Thermodesulfobacteria*, *Nitrospirae*, *Chlorobi*, *Gemmatimonadetes*, *Thermotogae* and *Synergistetes*; and 18 reads for 2012_GM_SE distributed over the aforesaid phyla except *Fusobacteria*, *Aquificae* and *Synergistetes*. **D**, Percentage distribution of total bacterial reads to the various phyla of the domain, as found by blastx against the *nr* protein database following methods described for **A**. **E**, Percentage distribution of total proteobacterial reads to the six classes of this phylum, as found by blastx against the *nr* protein database following same methods as **A** and **D**.

>1 Gb metagenomic sequence was generated for each GM_SE sample and used to draw synecological inferences (Table S1). As such, percentage distribution of metagenomic reads over various groups of a particular taxonomic category was considered as a direct measure of the relative abundance of those groups. Initially, reads were classified up to the domain level (Fig. 3A) by searching against the non-redundant (*nr*) protein database using the Organism Abundance tool of MG-RAST. Similarly, 16S rRNA gene (rDNA) reads within the datasets were classified by searching against the RDP database using the same tool (Fig. 3B). Read distribution patterns obtained from either analysis was broadly in agreement with each other. Unassigned and unclassified sequences constituted ~15% of every metagenome, while reads assigned to eukaryotes made up only 0.2–0.8% and those attributable to viruses were <0.05%. Archaeal contribution in the GM_SE metagenomes of 2010, 2011 and 2012 was 0.01%, 1% and 0.5% respectively.

Whatever little archaeal reads were there in the 2010 dataset were all attributable to *Euryarchaeota*. In contrast, out of the 1% archaeal share in the 2011_GM_SE metagenome, 0.37% came from *Thaumarchaeota*, 0.32% from *Euryarchaeota*, and 0.29% from *Crenarchaeota*. In 2012_GM_SE *Euryarchaeota* was the predominant phylum accounting for 0.23% of the total 0.5% archaeal share in the community. Out of the remaining 0.27%, *Thaumarchaeota* alone contributed 0.2%, while *Crenarchaeota* put in 0.07%. In both the 2011 and 2012 GM_SEs the thaumarchaeon *Nitrosopumilus* was the dominant genus accounting for 18–20% of all archaeal reads. Two other thaumarchaea *Cenarchaeum* and *Nitrososphaera*, alongside the *Euryarchaeota* member *Methanosarcina*, were the other dominant constituents of the 2011 and 2012 archaeal populations, collectively accounting for another 18% and 20% of all archaeal reads in the respective datasets. The exceptional hike in the proportion of *Crenarchaeota* in 2011_GM_SE was mainly due to increased representation of *Pyrobaculum*, which contributed ~50% of the all *Crenarchaeota* reads in this sample.

98–99% of the taxonomically classifiable reads of all three GM_SE editions were from *Bacteria*. Hence it was deemed appropriate to focus only on bacterial community dynamics for the rest of the study.

### Bacterial community dynamics in the GM_SE

A broad consensus was witnessed between the pictures of phylum-level read abundance emerging from comparison against the RDP (Fig. 3C) and the *nr* protein (Fig. 3D; Table S2) databases. As illustrated by these figures, the three GM_SE editions varied considerably in the relative abundance of the various bacterial phyla. The 2011 edition showed maximum heterogeneity, while 2010_GM_SE was the least diversified. *Proteobacteria* dominated all the three GM_SEs, but the extent of their predominance was exceptionally high in 2010_GM_SE where 95% of all reads were ascribed to this phylum. The only other phylum having >1% representation in 2010 was *Actinobacteria*. Again, within *Proteobacteria*, *Alphaproteobacteria* was always the most abundant class except in 2010_GM_SE where *Betaproteobacteria* predominated (Fig. 3E). There was a sharp decline in the abundance of *Proteobacteria* in 2011_GM_SE where ~41% of total classifiable reads were affiliated to the phylum. This was accompanied by remarkable upturns in the representation of *Chloroflexi*, *Bacteroidetes*, *Cyanobacteria*, *Firmicutes*, *Nitrospirae*, *Verrucomicrobia*, *Chlorobi*, *Deinococcus-Thermus*, *Acidobacteria* and *Planctomycetes*. Several marginal phyla, which did not have >1% representation in any of the three GM_SE metagenomes, also increased their abundance by ≥10 times (Fig. 2B; Table S2). Interestingly again, *Proteobacteria* rebounded in 2012, complemented by concomitant fall in the abundance of many of those phyla which had surged in 2011. Notably, *Actinobacteria* was the only phylum whose oscillation was in sync with this trend of *Proteobacteria*. On the other hand, most of the phyla that had increased their representation in 2011_GM_SE could not hold on to their gains in 2012. *Chloroflexi* and *Deinococcus-Thermus* were the biggest casualties of proteobacterial resurgence in 2012, suffering maximum losses and retreating almost back to their 2010 levels. *Cyanobacteria*, *Chlorobi*, *Nitrospirae*, *Gemmatimonadetes*, *Planctomycetes*, *Spirochaetes* and *Acidobacteria* also experienced considerable downturn between 2011 and 2012, but were still above their 2010 levels. *Firmicutes* suffered minimum cutback from their 2011 level, whereas only *Bacteroidetes* and *Verrucomicrobia* managed to further increase their representations in 2012.

**Figure 4:**
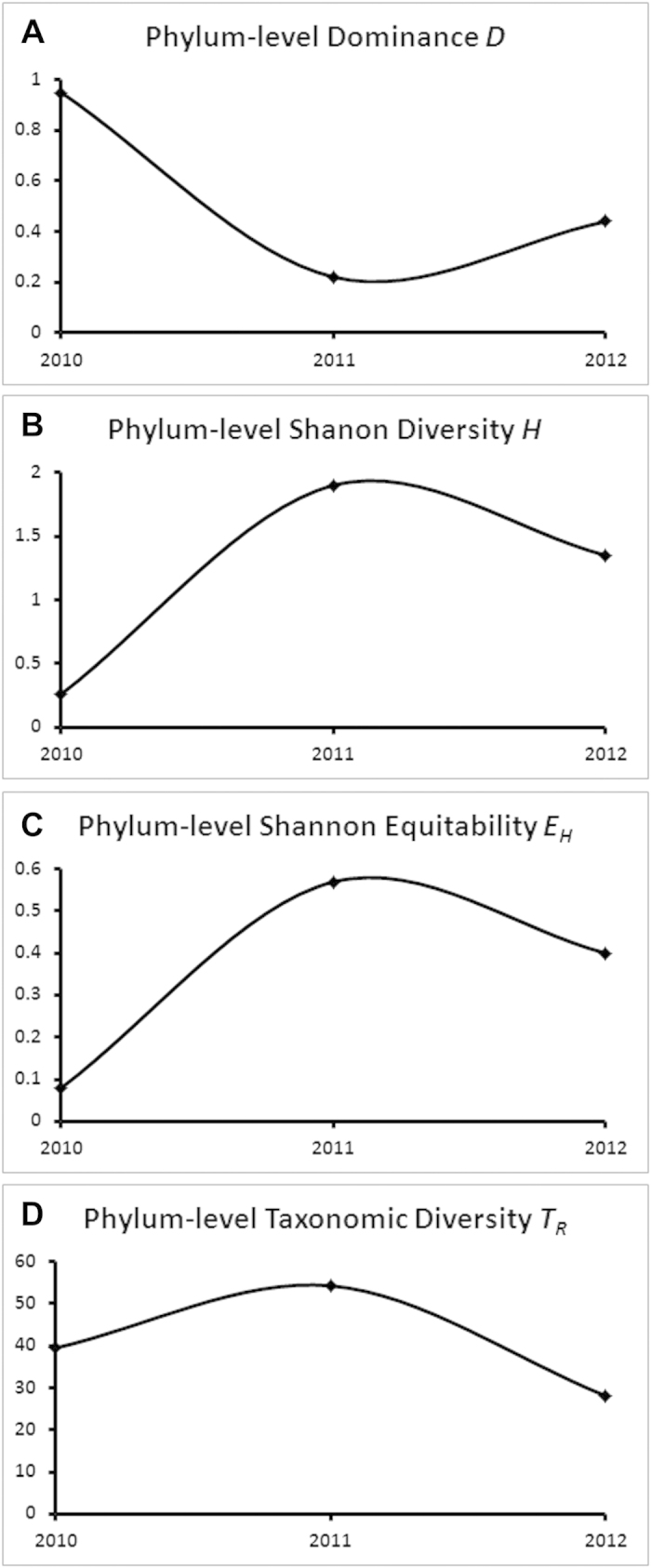
Mathematical indices comparing phylum-level ecological (A through C) and taxonomic (D) diversity of the three GM_SE community editions. **A**, **B** and **C** respectively shows the fluctuation of Dominance index *D*, Shannon diversity index *H*, and Shannon equitability index *E*_*H*_ over the three assessment years calculated from metagenomic sequence data. ****D**** shows the trends observed for the new Relative Taxonomic Diversity Index *T*_*r*_ calculated from amplified 16S rDNA sequence data.

Different mathematical indices were calculated (Table S3 A through S3 C) to quantify the phylum-level ecological diversity (which involves ‘what types’ as well as ‘how many’) of the GM_SEs. Corroborating the relative abundance trends of Fig. 3, Simpson dominance index *D* was exceptionally high for 2010_GM_SE (0.95), moderate for 2012_GM_SE (0.44), and lowest for 2011_GM_SE (0.22) (Fig. 4A). Expectedly, fluctuations of Shannon diversity index *H* (Fig. 4B) and Shannon equitability index *E*_*H*_ (Fig. 4C) were inverse to the trend observed for *D*.

### Concurrence between genus- and phylum-level trends

**Figure 5:**
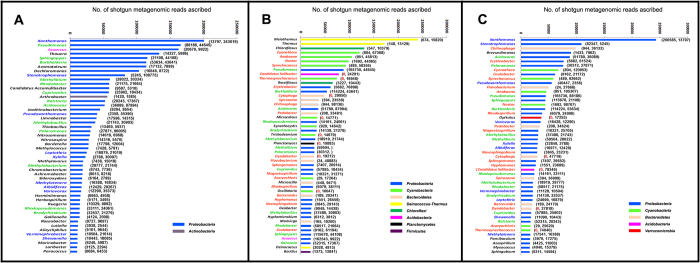
Comparison of histograms for the 50 most-abundant genera identified in the three GM_SE metagenomes. **A** through **C** represents histograms for 2010 through 2012 GM_SE editions, in that order. Colors of the histogram bars indicate phylum-level affiliation of the genera reported. Genus names in green font are common to all the three GM_SEs; genus names in blue font are common only to the 2010 and 2012 GM_SEs; those in red font are common only to the 2011 and 2012 GM_SEs; the sole name common only to 2010 and 2011 GM_SE is in purple font. The genera that are unique to any one GM_SE edition are in black font. The two consecutive numbers given in parenthesis next to a genus bar in ‘**A**’ represent the number of reads ascribed to that genus in 2011_GM_SE and 2012_GM_SE respectively. Similarly, the two numbers in parenthesis next to a genus bar in ‘**B**’ stand for reads ascribed to that genus in 2010_GM_SE and 2012_GM_SE respectively, while similar numbers in ‘**C**’ represent reads ascribed to a given genus in 2010_GM_SE and 2011_GM_SE respectively.

It was remarkable to observe that >50% of all taxonomically classifiable reads of any given GM_SE metagenome were assigned to the 50 most-abundant putative genera identified in that metagenome (Fig. 5). In 2010_GM_SE, top-50 genera accounted for ~80% of all classifiable reads, thereby justifying its high dominance index *D*. In 2011 and 2012 GM_SE respectively top-50 genera accounted for 56% and 58% of all classifiable reads. Interestingly again, year-on-year constancies and fluctuations exhibited by the top-50 genera mirrored the trends observed for the relative abundance of GM_SE phyla in Fig. 3D. As a mark of stability, 13 names (written in green font in Fig. 5) were common to all the three top-50 generic lists (referred to hereafter as the ‘leaderboard’). The leaderboard of 2010_GM_SE (Fig. 5A) encompassed representatives from only two phyla, while that of 2011_GM_SE (Fig. 5B) was taxonomically most inclusive (encompassing genera from eight phyla), followed by that of 2012_GM_SE that included genera from five different phyla (Fig. 5C). These data also corroborated the synecological indices of the three GM_SE editions.

In 2010_GM_SE, all but only one genus in the top-50 were from *Proteobacteria*. Apart from the 13 genera common to all three leaderboards and the single genus *Azoarcus* that was common to the 2010 and 2011 GM_SEs, the rest of the 36 members of the 2010_GM_SE leaderboard dropped from the corresponding list of 2011. Interestingly, 10 out of these 36 (names in blue font in Fig. 5) reappeared in the 2012_GM_SE leaderboard. Moreover, none of those 36 leaderboard members of 2010_GM_SE which dropped from the list in 2011 (including those 26 which failed to make a comeback in 2012) were actually dispelled from the community. The fact that they continued to occupy significant positions in the 2011 and 2012 GM_SEs was evident from their high read counts in these two metagenomes (see the numbers given in parenthesis in Fig. 5A).

2011_GM_SE had only 23 *Proteobacteria* among its top-50 genera. Concomitant to this proteobacterial retreat there were 36 new entries in the 2011 leaderboard, out of which, 21 remained predominant in 2012_GM_SE (names in red font in Fig. 5). Again, among the 36 new leaders of 2011 only eight were completely absent in the 2010_GM_SE metagenome (see names in Fig. 5B whose first number in parenthesis is zero). Again, all these eight newcomers maintained significant presence in 2012_GM_SE, with four of them even managing to retain their place in that leaderboard.

Proteobacterial resurgence was again conspicuous in 2012_GM_SE as 35 genera affiliated to the phylum hit the leaderboard. Concomitantly, all genera from *Deinococcus-Thermus (three)*, *Chloroflexi* (two), *Planctomycetes* (one) and *Firmicutes* (one), which had made it to the top-50 in 2011_GM_SE, were relegated from the chart. Number of genera from *Cyanobacteria* and *Bacteroidetes* in the top-50 list also decreased in 2012_GM_SE. Additionally, there were six new leaderboard entrants in 2012_GM_SE, all of which except *Opitutus* were already there in the community since 2010 but gained preeminence only in 2012.

These numbers highlighted the resilience of the dominant components of the GM_SE, even as the community remained consistently receptive towards newcomers, which in their turn got stably incorporated into the guild. The last inference was evidenced by the fact that despite several transpositions in and out of the top-50 list over the three assessment years, not a single instance was apparent where an erstwhile leaderboard member went totally undetected in a subsequent GM_SE edition (follow the numbers in parentheses of Fig. 5).

### Correlating populational and environmental fluctuations

It is presumable that in between 2010 and 2011 disappearance of the PT-top geyser and desiccation of the substratum ushered wide-ranging ecological limitations for the GMMC and eventually rendered irreparable damage to its habitat. In this scenario, even if a substantive fraction of the 2010 community managed to render dispersal to the southern slope, this calamity must have already impacted the community’s structure and function(s) significantly. In course of time, the distinct physicochemical conditions of the newfound habitat ought to have impacted the community further. However, it must be acknowledged that we lacked data on the environmental constraints, or for that matter, community alterations within the degenerating mats, around the time points when the west slope environment was actually drying up. As such, direct impact assessment of habitat degradation was not possible and environment-community correlations could only be based on comparative geochemistry and metagenomics of the spatiotemporally discrete hot water outflows and mat samples respectively. On top of that, comparative physicochemical information (see Table 1 or Fig. 2A) used in the correlation studies were averages of data collected at three discrete time points of only one particular day. Hence there was no data in hand to prove that the apparent year-on-year geochemical variations between the niches did not also occur on a more regular, say monthly or seasonal, basis.

**Figure 6:**
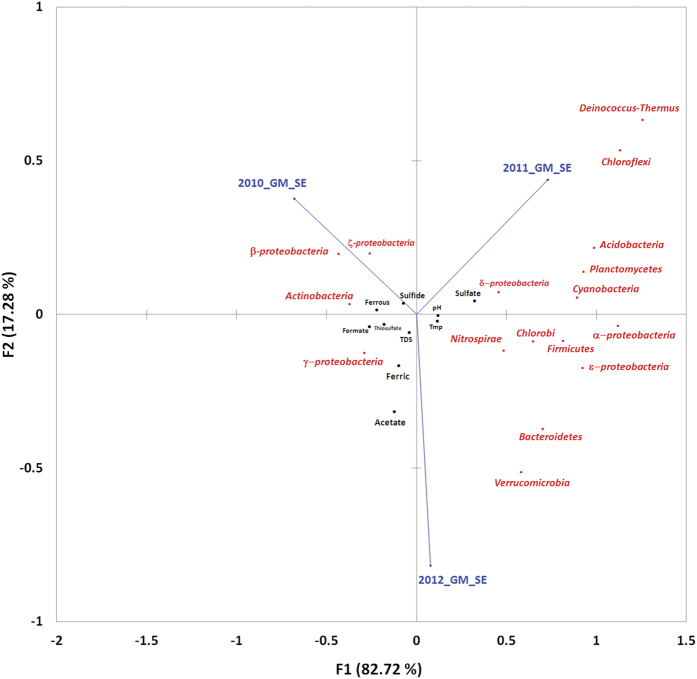
Canonical correspondence analysis of the bacterial community structure of the three GM_SE editions based on the relative abundance (metagenomic read affiliation) of constituent phyla. Only for *Proteobacteria*, abundance data were resolved up to the class level. Distance of the black dots from the origin denotes environmental variables; distance of the red spots from the origin denotes relative abundance of the phylum/class, while blue dots denote the three samples analyzed.

The above caveat notwithstanding, it is noteworthy that nine out of the ten tested physicochemical parameters of the SE outflow showed identical patterns of oscillation over the three assessment years. Sulfate concentration was the only exception to the U-shaped curves observed for the nine other factors. Correspondingly, all the GM_SE phyla except *Proteobacteria* (particularly *Gammaprotoebacteria*) and *Actinobacteria* exhibited bell-shaped trends of population fluctuation over the three assessment years (see Fig. 2B & 3E). Temperature, pH, total dissolved solid (TDS), and concentrations of thiosulfate, sulfide, Fe^2+^ and Fe^3+^ iron, acetate and formate, all were at their relative highs in 2010. Next year all these values dipped sharply, only to increase again in 2012. While in 2012 most of these parameters were still below their 2010 levels, ferric iron recovered to its 2010 high while acetate went past that level. As such, the physicochemical milieu of 2011_GM_SE was quite different from that of 2010_GM_SE, while environmental parameters for 2012_GM_SE tended partly back towards the 2010 values. Here it may be recalled that a wide divergence between 2010_GM_SE and 2011_GM_SE, and an intermediary status of 2012_GM_SE, was already evident from community structure comparisons. These prima facie impressions were quantitatively established when the environmental conditions experienced by the three GM_SE editions were statistically correlated with their community compositions. Bray-Curtis similarities and Euclidean distances between GM_SE sample pairs (Table S4) evidenced the wide divergence between 2010 and 2011 GM_SEs and the intermediary status of 2012_GM_SE, while Canonical Correspondence (CCA) (Fig. 6) and Principal Coordinate (PCoA) (Figure S2) analyses with phylum/class-level data graphically illustrated the same. CCA further revealed that the trifurcated relationship of the three GM_SEs stemmed mainly from the concentration of *Betaproteobacteria* in 2010_GM_SE, and *Deinococcus*-*Thermus* and *Chloroflexi* in 2011_GM_SE, while there was no such skewed population in 2012_GM_SE. Besides such large-scale population growths, a few instances of modest but significant population expansion were also apparent, e.g. (i) *Actinobacteria* and *Zetaproteobacteria* in 2010_GM_SE, (ii) *Acidobacteria*, *Deltaproteobacteria*, *Cyanobacteria* and *Planctomycetes* in 2011_GM_SE, and (iii) *Verrucomicrobia* and *Bacteroidetes* in 2012_GM_SE. Separation of the three GM_SE editions in CCA was further explained by the fact that some environmental factors were exceptionally high or low for certain particular samples. Such examples included (i) higher sulfide and Fe^2+^ in the 2010 outflow, (ii) lower pH and temperature alongside higher sulfate in 2011, and (iii) higher acetate in 2012.

As observable in Fig. 6, several cases of population fluctuation in the GM_SE were correlated to the oscillations of one or more environmental factors. In the following section we discuss only those cases which were further corroborated by strong Pearson correlation values, i.e. *r* numerically >0.8 alongside *P* < 0.05. Fluctuations in the abundance of *Deltaproteobacteria*, *Acidobacteria* and *Gammaproteobacteria* vis-à-vis sulfur species concentrations and pH were some of the most conspicuous correlations observed in these analyses. Decrease in the pH of the SE outflow in 2011 coincided with ~50% increase in sulfate concentration. These were accompanied by matching decreases in the flow rate and, sulfide and thiosulfate content of the fluid (Table 1). On the contrary, the 2012 pH upturn towards neutrality concurred with ~22% slump in sulfate content alongside relative hikes in flow rate, sulfide and thiosulfate content. However, none of these reversals were high enough to take the concerned parameters back to their 2010 levels. Within this gamut of fluctuations, increase in sulfate concentration of the 2011 SE outflow could have particularly ushered the five and a half fold increase in the relative abundance of *Deltaproteobacteria* in 2011_GM_SE (Fig. 3E), which in its turn was mainly attributable to rise in read counts of sulfate-reducing *Desulfuromonadales* and *Desulfovibrionales* members such as *Geobacter*, *Pelobacter*, *Desulfovibrio*, etc. The fact that deltaproteobacterial abundance in 2012_GM_SE, despite being halved from its 2011 level, still remained more than twice the level of 2010_GM_SE could be attributed to the moderately high sulfate concentration of 2012. It was further noteworthy that upturns in the abundance of sulfate-reducing bacteria coincided with the steep population growth of *Cyanobacteria*. This observation was in agreement with earlier reports of syntrophism between the two groups within microbial mats, where cyanobacterial excretory products (such as glycolate) are used by sulfate-reducing bacteria as electron sources^9^. The *Acidobacteria* population oscillated (Fig. 2B) in sync with *Deltaproteobacteria* (Fig. 3E), even as the former’s fluctuations were sharper. While both the groups showed strong positive and negative correlations with sulfate concentration and pH respectively, the small dip in the pH of the OFC could have driven the influx and/or proliferation of *Acidobacteria* in 2011_GM_SE. *Acidobacteria* can slow down metabolism and live long under low-nutrient conditions and substratum desiccation^10^. This together with their diverse mechanisms of respiration may have helped them overcome similar adversities between 2010 and 2011. The gammaproteobacterial population oscillated in a pattern opposite to that of *Deltaproteobacteria* and *Acidobacteria*, thereby showing strong positive and negative correlations with thiosulfate and sulfate respectively. These data were consistent with the fact that decrease in the overall population of *Gammaproteobacteria* in 2011_GM_SE involved sharp decrease in lithotrophic sulfur-oxidizing bacteria (SOB) like *Thioalkalivibrio*, *Acidithiobacillus*, *Allochromatium* and *Halothiobacillus*. *Actinobacteria* was the only phylum other than *Proteobacteria* that showed U-shaped oscillation over the years. Its abundance correlated positively with Fe^2+^, which was remarkable since iron oxidation is reportedly not widespread in *Actinobacteria*, and that too mostly confined to *Acidimicrobidae*^11^, a subclass absent from the *Actinobacteridae*-dominated PT GM_SE.

**Figure 7:**
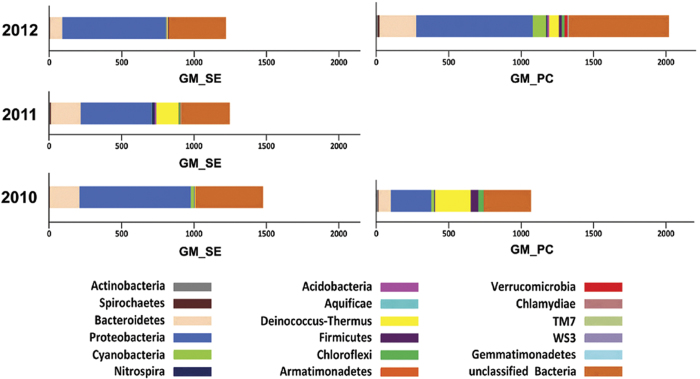
Phylum-level distribution of the bacterial OTUs of the three GM_SE and two GM_PC communities. Note that there was no GM_PC complement in 2011.

Other significant canonical correspondences and linear correlations observed in the GM_SE community dynamics included (i) *Cyanobacteria* versus pH, TDS and formate (*r* = −0.998 for all three); (ii) *Firmicutes* versus sulfide and ferrous (*r* = −0.999 and *r* = −0.998 respectively); (iii) *Nitrospirae* versus pH, TDS and formate (*r* = −1.000 for all); and (iv) *Planctomycetes* versus pH, TDS and formate (*r* = −1.000 for all); (v) *Deinococcus-Thermus* versus temperature and ferric iron (*r* = −0.997 for both); and (vi) *Chlorobi* versus sulfide (*r* = −1.000). Negative correlations between *Deinococcus-Thermus* and temperature, and *Chlorobi* and sulfide, appeared strange. However, subsequent 16S rDNA sequence-based diversity estimation of the GM_SEs versus the GM_PCs (Fig. 7) showed that the growth of *Deinococcus-Thermus* in 2011_GM_SE could be due to their dispersal from the 2010_GM_PC. Again relative abundance of *Chlorobi* or green sulfur bacteria (GSB) was lowest in 2010_GM_SE (Fig. 2B) when sulfide concentration in the OFC was at its highest (Fig. 2A). Unusually again, the GSB population increased to its highest level in 2011_GM_SE when sulfide was at its lowest. This could be explained by fact that *Chromatiaceae* and *Ectothiorhodospirilaceae*, or the purple sulfur bacteria (PSB), accounted for >10 times, 1.25 times and 1.4 times more reads than *Chlorobi* in the metagenomes of 2010, 2011 and 2012 GM_SE respectively. Now, despite their common requirement for sulfide, the PSB are nutritionally more versatile than the GSB by virtue of their ability to utilize organic compounds and also grow chemotrophically on thiosulfate in the dark by reducing oxygen^12^. So in 2010_GM_SE, the PSB might had outnumbered the fastidious GSB by making full use of thiosulfate (that was also abundant that year) and then by virtue of their numbers usurped the available sulfide also. However, with thiosulfate going down next year, the PSB potentially lost this advantage, thereby allowing the GSB to avail whatever sulfide was available in the 2011 discharge and grow from ~0.1% of all bacteria in 2010_GM_SE to ~0.5% in 2011_GM_SE.

### Constancy and variability of metabolic types in the GM_SE metagenomes

Detection of photosynthetic genes in all GM_SE metagenomes concurred with their hyaline to deep green appearance. As such, the detectable repertoire of genes involved in electron transport and photophosphorylation, and chlorosome and phycobilisome light-harvesting complexes was higher in 2011 and 2012 GM_SEs than in 2010_GM_SE. Notably again, photosynthetic genes detected in 2010_GM_SE were mostly proteobacterial in origin, while those of the next two editions were mainly from *Cyanobacteria*.

Invariable presence of multiple homologs of all the *sox* (sulfur oxidation) structural genes of chemo- as well as photo-lithotrophic bacteria in all the three GM_SE metagenomes indicated that reduced sulfur species, which were by and large abundant in this spring system, could be a key source of energy and/or electron for the community. Molecular hydrogen could also be an important source of energy and/or electron for the autotrophic as well as mixotrophic members of the GM_SE. This was suggested not only by the detection of energy-generating NAD^+^ -reducing [NiFe] hydrogenases in all three GM_SE metagenomes but also by the occurrence of the hydrogen-oxidizing “Knallgas” bacterium *Ralstonia* in all the three lists of top-50 genera (Fig. 5). Other such organisms as *Rhodococcus*, *Azotobacter*, *Chloroflexus* etc., which too can utilize H_2_ as a source of energy and/or electron via the oxyhydrogen “Knallgas” reaction 2H_2_ + O_2_ → 2H_2_O^13^,^14^ were also significantly represented in all these metagenomes. Consistent presence of facultatively hydrogen-oxidizing chemolithoautotrophic diazotrophs like *Bradyrhizobium*, and phototrophs like *Rhodobacter* and *Rhodopseudomonas*^14^, in significant proportions further underscored the potential importance of hydrogen as an energy source of the community. Besides sulfur- or hydrogen-based lithotrophy, anaerobic carbon monoxide (CO) oxidation could also be another important source of energy and electron for the GM_SE. This was evidenced by the constant predominance of the anoxygenic phototroph *Rhodospirillum* [which can grow anaerobically in the dark on CO as the sole source of energy^14^] and the presence of several homologs of CO dehydrogenase (CODH) and CO-insensitive [NiFe] hydrogenases in all GM_SE metagenomes. Notably, other carboxydotrophs like *Streptomyces*^15^,^16^, *Carboxydothermus* and *Oligotropha*^14^ also accounted for significant fractions of metagenomic reads in all GM_SE editions.

Hydrothermal supply of iron (particularly Fe^2+^) could be another key determinant of the GM_SE community structure. While an apparent parallelism existed between Fe^2+^ concentration in OFCs (Table 1, Fig. 2A) and the iron richness of the GM_SE substrata (Figure S3), fluctuations of these two factors correlated well with the oscillation of iron-oxidizing bacteria (IOB) within the community (see lower panel of Figure S3). In 2010 the hydrothermal discharge and the deposited sinters were remarkably rich in Fe^2+^ and Fe^3+^ respectively (Table 1). Correspondingly, there were at least eight lithotrophic or organotrophic IOB (viz. *Pseudomonas*, *Rhodobacter*, *Thiobacillus*, *Leptothrix*, *Sideroxydans*, *Rhodopseudomonas*, *Gallionella* and *Marinobacter*)^17^ in the list of top-50 genera of 2010_GM_SE. Out of these eight genera three were also present in the corresponding list of 2011_GM_SE, while four made the cut in 2012_GM_SE (Fig. 5). IOB, as such, accounted for >10% of all bacterial metagenomic reads of 2010_GM_SE, while their read share was ~3% in the next two GM_SE editions. Drop in IOB abundance in the GM_SE clearly coincided with the sharp decline in Fe^2+^ availability in 2011, but, due to some unknown reason, their population did not recover appreciably in 2012 despite considerable increase in Fe^2+^ abundance in the discharge as well as the substratum.

Significant synchrony was also observed between the oscillation of the *Shewanella* population (Fig. 5) and that of iron, thiosulfate or TDS in the OFC (Fig. 2A), or for that matter, iron in the substratum (Figure S3). Under oxygen-stressed conditions *Shewanella* respire by reducing diverse oxidized metals and other substrates like fumarate, nitrate, trimethylamine *N*-oxide, dimethyl sulfoxide, sulfite, thiosulfate and elemental sulfur^18^. As such, this facultatively aerobic alphaproteobacterium, or its close phylogenomic relatives, could complement the biogeochemical roles of IOB and SOB and help complete the iron and sulfur cycles in the PT slope environment. They could also play central role in the biomineralization process in the slope microfacies by reducing dissolved metal ions from the hydrothermal fluid to their insoluble sulfide or oxide forms.

So far as carbon sources were concerned, all three GM_SE metagenomes encompassed large number of CO_2_ fixation-related genes such as those involved in carboxysome, CO_2_ uptake, Calvin-Benson cycle, and photorespiration/oxidative C2 cycle. Potentials for autotrophic acetogenesis via the acetyl-CoA “Wood-Ljungdahl” pathway^19^ were noticeable in the GM_SE throughout the assessment period. However, standard acetogens like *Clostridium*, *Moorella*, *Eubacterium* and *Thermoanaerobacter* were present in significant proportions only in the 2011 and 2012 editions of the GM_SE, but not in 2010_GM_SE. As such, these four genera together accounted for 0.01%, 0.34% and 0.27% of all taxonomically classifiable reads of the 2010, 2011 and 2012 GM_SE metagenomes respectively. Nonetheless, genes for some of the key enzymes of the bacterial acetyl-CoA pathway, viz. (i) formate dehydrogenase, (ii) methenyltetrahydrofolate cyclohydrolase and methylenetetrahydrofolate dehydrogenase complex, and (iii) methylenetetrahydrofolate reductase were present in all GM_SE metagenomes, including the 2010 edition. This observation illustrated that in 2010 cryptic potentials for acetogenesis could have well been dispersed up to the SE community, even as the main hub of acetogens lied somewhere up in the reductive gradient closer to the venting point. On the other hand, although both 2011 and 2012 GM_SE had equally increased abundance of acetogenic genera, genes for the three sub-units of key acetogenesis enzyme acetyl CoA synthase^19^, viz. (a) acetyl-CoA synthase corrinoid iron-sulfur protein; (b) CO dehydrogenase/acetyl-CoA synthase, CO dehydrogenase subunit; and (c) CO dehydrogenase/acetyl-CoA synthase, acetyl-CoA synthase subunit, appeared only in the 2012_GM_SE metagenome, plausibly due to sheer stochasticity. Anyway, these observations certainly implied that the altered SE conditions of 2011, which appreciably lingered until 2012, promoted the growth of acetogenic bacteria. Now this population growth could have been driven by elements that were already there in subdued abundance in 2010_GM_SE and/or those which migrated from more reduced and hotter niches of 2010.

Remarkably again, the apparent rise of acetogens in 2011 as well as their significant retention through 2012 coincided with identical population dynamics of methanogens and sulfate-reducing bacteria, which like the acetogens also employ the Acetyl-CoA pathway either in the direction of acetate/biomass synthesis or that of acetate degradation^19^. In this connection it is noteworthy that the summation of potential methanogens, acetogens and sulfidogens (sulfate-reducers) did not account for more than 5% of total metagenomic reads in any GM_SE sample, but their steep rise between 2010 and 2011 (followed by strong retention through 2012) was relatively significant. For example, the representation of methanogens went up from <0.01% of all classifiable reads in 2010_GM_SE to 0.21% in 2011_GM_SE and 0.14% in 2012_GM_SE, corresponding to which 6, 10 and 16 different methanogenesis-associated genes were identified in the respective metagenomes (Table S5). Similarly, proportion of potential sulfidogens (which included probable members from *Deltaproteobacteria*, *Firmicutes* and *Nitrospirae*) increased from 1.25% of all reads in 2010_GM_SE to almost 4.0% in 2011 and 3.3% in 2012. As envisaged for the acetogens, the observed upturn of methanogenic and sulfidogenic populations could have also been driven by elements already present in 2010_GM_SE (albeit, as minuscule minorities) and/or by immigrants from more reduced and hotter niches of 2010 (as apparent from subsequent analyses). However, simultaneous rise of these three biotypes in the same SE niche was bioenergetically intriguing since all the three metabolisms are hydrogen-requiring processes and the populations in question ought to compete with each other for hydrogen. Sulfate-reducing bacteria are known to utilize H_2_ at concentrations lower than that required by methanogens and their ability to outcompete the methanogens plausibly arise from the more-positive reduction potential of SO_4_^2−^ than that of CO_2_^20^. From this point of view, the spike in methanogenic archaea in 2011_GM_SE was all the more intriguing because it happened when sulfate and sulfide contents of the SE outflow were at their three-year highest and lowest levels respectively. In other oxygen-stressed environments with sufficient quantities of SO_4_, H_2_S is reportedly the predominant reduced product, and the major fate of biodegradable organic carbon is oxidation to CO_2_ (formed from the CH_3_ group of acetate)^20^. Conversely, CH_4_ replaces H_2_S as the main reduced product typically when SO_4_ is limiting (as organic carbon is disproportionated to CO_2_ and CH_4_ via H_2_- or formate-using aceticlastic reactions, which form CH_4_ from the CH_3_ of acetate)^20^. As such, the unusual expansion of the GM_SE methanogenic population amidst copious sulfate availability could have been rendered feasible by metabolisms based on methylated substrates like methylamines, which are monopolistic substrates rapidly fermentable (to CH_4_, CO_2_ and NH_3_) by methanogens. So far as the supply of trimethylamine is concerned, it can form from betaine glycine or other related osmoprotectants^20^, which in their turn are likely to be produced profusely by geothermal bacteria to balance the osmolarity of their cytoplasm with that of the thermal fluid^21^,^22^. Predominance of *Methanosarcinaceae* among the GM_SE methanogens in tandem with the steady detection of methylamine fermentation genes (monomethylamine permease, and trimethylamine:corrinoid and methanol:corrinoid methyltransferases) insinuated the prevalence of methylotrophic methanogenesis in the community. Nevertheless, since the observed oscillation trend of acetate in the thermal discharge was opposite to that of acetogenic/methanogenic populations in the GM_SE it was quite likely that aceticlastic reactions were also rendered significantly. All these circumstantial evidences collectively hinted that H_2_ was a limiting factor for the community, which again could plausibly be compensated by the formate (an equally efficient reductant for methanogens) available in the system (Table 1). Anyway, even if the methanogens were out of the race for H_2_, the number of potential H_2_-utilizing processes and the count of corresponding bacteria were still significantly high in the GM_SE. As such, the community ought to have ensured sufficient and steady supply of H_2_, either from the native H_2_-generating bacteria and/or by inorganic reaction between Fe^2+^ and water.

So far as biotic H_2_ contribution is concerned, phototrophic *Proteobacteria* like *Rhodobacter* and *Rhodospirillum*, perpetually predominant in the GM_SE, could transform carbon substrates like lactate, acetate, butyrate, malate, etc. (using dinitrogen, glutamate or aspartate as nitrogen source) to CO_2_ and H_2_, which in their turn could also be reused for photoautotrophic growth^23^. Fermentation of organic matter could be another major energy-yielding as well as H_2_-forming process in the community. Constant occurrence of several genes for [NiFe] hydrogenases and formate hydrogenlyase indicated that mixed-acid fermentations could be in vogue in the GM_SE. Cyanobacteria like *Cyanothece* and *Microcoleus* and anoxygenic phototrophs like *Rhodospirillum*, *Rhodobacter* and *Rhodopseudomonas* (which ferment endogenous reserves in the dark to produce H_2_ as one of the fermentation products) could significantly contribute to H_2_ production via this route. Chemotrophic CO oxidation by anaerobic anoxygenic phototrophs like *Rhodospirillum* could be another viable source of molecular hydrogen in the GM_SE. Detection of CODH and CO-insensitive [NiFe] hydrogenase (which together catalyze the net reaction CO + H_2_O → CO_2_ + H_2_) in all three metagenomes spoke volubly in favor of this process. H_2_ could also be produced substantively as a byproduct of nitrogenase-mediated N_2_ fixation without involving a hydrogenase^14^. This was insinuated not only by the abundant dinitrogen-fixing rhizobia but also the various nitrogen-fixation genes detected in all three GM_SE metagenomes. Last but not the least; metagenomic data also suggested that alkaline phosphatase-mediated phosphite oxidation (H_3_PO_3_ + H_2_O → H_3_PO_4_ + H_2_) could also be a viable source of H_2_ in the GM_SE.

Besides a large number of heterotrophic bacteria capable of growing on complex sugars and amino acids, several methylotrophic and methanotrophic genera^24^ predominated the GM_SE consistently. Although the concentration of methylated compounds in the PT environment was not measured their importance as carbon source for the community was apparent from the occurrence of *Methylibium*, *Methylobacillus*, *Methylobacterium*, *Methylotenera*, *Methylovorus* and *Methylococcus* in the top-50 genera list of all three metagenomes (see Fig. 5 and Table S6). In addition, all the GM_SE metagenomes encompassed genes for at least one of the three subunits of methane monooxygenase, the key enzyme oxidizing the C-H bond of methane and other alkanes^25^.

### Taxonomic diversity flux corroborated the fluctuations in ecological diversity

To get a precise picture of the taxonomic diversity (which only involves ‘what types’ and not ‘how many’) of the discrete GM_SE editions we estimated their species richness by analyzing amplified 16S rDNA fragments. V3 regions of all potential bacterial 16S rDNA present in a GM_SE metagenome was PCR-amplified using *Bacteria*–specific primers, but no amplicon could be obtained with *Archaea*–specific primers plausibly due to their extremely low abundance. The bacterial V3 amplicon pools were sequenced by Ion PGM up to such depths which ensured that plateaus of rarefaction curves were reached. Table S7 documents all data pertaining to the clustering of OTUs from the PGM reads.

The GM_SEs of 2010, 2011 and 2012 encompassed 1478, 1248 and 1220 OTUs respectively (Fig. 7). The species richness of 2010_GM_SE, though numerically highest among the three GM_SEs, was confined to only a few higher-level groups. In contrast, 2011_GM_SE was taxonomically most dispersed, followed by 2012_GM_SE. As such, the classifiable OTU diversity of 2010, 2011 and 2012 GM_SE was distributed over 9, 16 and 11 phyla respectively (Fig. 7, Table S8). Likewise, 12, 22 and 14 classes (Table S9), and 19, 52 and 27 genera (Table S10) were identifiable in 2010, 2011 and 2012 GM_SE in that order. These numbers were suggestive of an oscillation in the GM_SE taxonomic diversity in line with the trend observed for its ecological diversity. They further highlighted that higher OTU count did not necessarily mean greater taxonomic diversity if the spread of the OTUs over various higher-level taxa was included in the concept of diversity. However, since in the existing literature there is no mathematical scale to quantify the taxonomic spread of OTU sets we first had to formulate such an index and then use it to analyze potential swings in the GM_SE alpha diversity. It needs to be clarified at this juncture that this new Relative Taxonomic Diversity Index (*T*_*r*_) only gives a comparative measure of the alpha diversities of closely related communities at a chosen hierarchic level (‘phylum’ in the present case) and is not an absolute measure of taxonomic diversity of a community. As such, it can only quantify the taxonomic diversity of a community relative to other spatially- or temporally-linked communities.

So far as calculating *T*_*r*_ was concerned, first, diversity pertaining to the i^th^ phylum within a given GM_SE edition (p_i_) was estimated as a proportion of the cumulative diversity of that phylum encompassed by all three GM_SEs. Equation 1 gives the general expression for this ratio. Notably, the denominator (N_i_) of the term p_i_ represented the taxonomic scope of the i^th^ phylum in the whole GM_SE system, and was determined as follows: (i) First, the three multifasta files that respectively contained the consensus sequences of all the OTUs of the three GM_SE editions were merged into a single multifasta. (ii) Pair-wise alignment of all the sequences of this merged file was performed, followed by their tree-based clustering at 97% sequence similarity level. The new OTU superset thus formed gave the full scope of species diversity in the whole system. (iii) Finally, the consensus sequences in this OTU superset were classified via the RDP Classifier, and those OTUs which were found to be affiliated to the i^th^ phylum gave the taxonomic scope (N_i_) of that phylum in the whole GM_SE system. After p_i_ value was calculated for any phylum identified in a given OTU set it was multiplied by the square roots of their corresponding n_i_ value to account for the actual species count for that phylum. The resulting products were then summed across phyla, and the summation gave the final *T*_*r*_ value for the OTU set or GM_SE edition in question (equation 2).


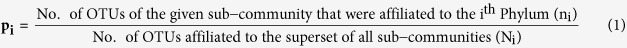



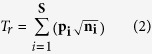


All raw calculations involved in the current analysis are given in Table 2. As such, *T*_*r*_ value for 2011_GM_SE happened to be the highest, i.e. 54.3 out of a maximum possible 101.85, which was achievable only if a community edition by itself encompassed the whole diversity scope of the community system. 2012_GM_SE had the lowest *T*_*r*_ value (28.1), while 2010_GM_SE (39.51) enjoyed intermediate status. Thus, the year on year fluctuations in the *T*_*r*_ value of the GM_SE (Fig. 4D) almost mirrored the oscillations witnessed for the ecological diversity indices *H* and *E*_*H*_. The only distinction was that the *T*_*r*_ of 2012_GM_SE was less than that of 2010_GM_SE. Although the *T*_*r*_ values suggested an eventual loss of taxonomic diversity in the GM_SE, subsequent inclusion of the two GM_PC samples in a comprehensive OTU analysis of the entire PT GMMC revealed that a considerable portion of taxonomic diversity concentrated in 2011_GM_SE actually migrated to the PC niches in 2012 rather than being lost from the system.

### Constancy, resilience and flux of bacterial groups in the entire GMMC

**Figure 8:**
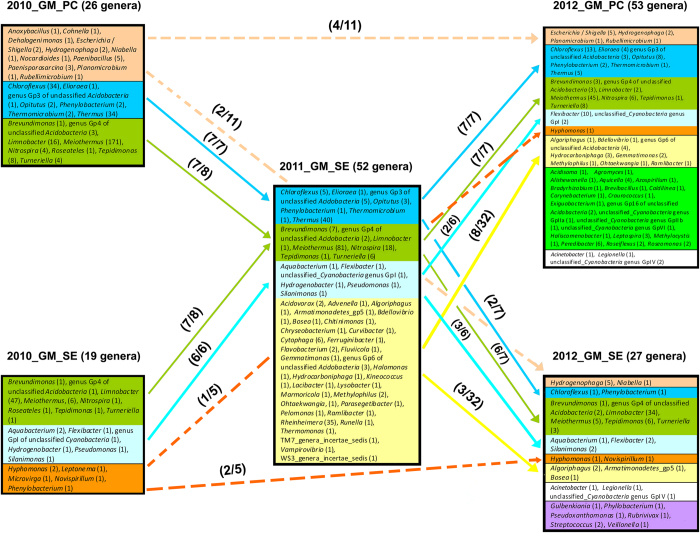
Genus-level distribution of bacterial OTUs identified in the five GMMC samples and plausible spatiotemporal transmissions of the genera. Numbers in parenthesis beside generic names indicate the number of OTUs identified under the genus in the given sample. The 2010 GMMC encompassed 7 such GM_PC-specific genera (names shaded deep blue) which could be re-identified in 2011_GM_SE; subsequently all these were rehabilitated to 2012_GM_PC even as 2 remained conserved in 2012_GM_SE (paths traced by deep blue arrows). 11 other GM_PC-specific genera of 2010 (shaded crimson) went missing from 2011_GM_SE; however, 4 and 2 of them resurfaced in 2012 GM_PC and GM_SE respectively (dotted crimson arrows). Similarly, 6 genera unique to 2010_GM_SE (shaded light blue) could all be re-identified in 2011_GM_SE; these were partitioned 2 apiece in 2012 GM_PC and GM_SE (light blue arrows). 5 other genera unique to 2010_GM_SE (shaded orange) went missing from 2011_GM_SE; however, 1 and 2 OTUs out of 5 respectively resurfaced in 2012 GM_PC and GM_SE (dotted orange arrows). Out of the 8 genera common to 2010_GM_PC and 2010_GM_SE (shaded deep green) 7 were conserved in 2011_GM_SE; out of these 7, all were present in 2012_GM_PC, while 1 went missing from 2012_GM_SE. 32 new genera (shaded yellow) were added to the GMMC in 2011, out of which only 8 and 3 were re-identified in 2012 GM_PC and GM_SE respectively (yellow arrows). The 2012 GMMC inducted 22 (names shaded bright green) and 6 (names shaded lavender) totally new genera via the GM_PC and GM_SE windows respectively. 3 more new genera were also inducted in 2012 via both GM_PC and GM_SE.

**Figure 9:**
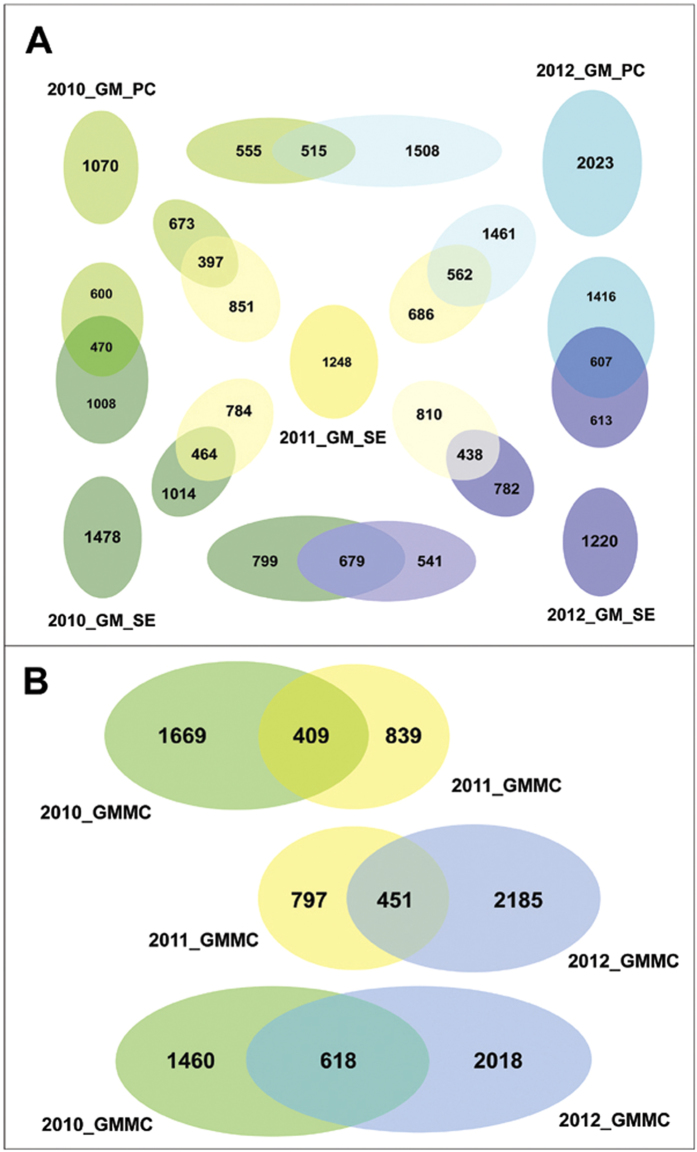
Number of unique and shared OTUs between pairs of GM_PC and GM_SE samples (A) as well as pairs of total GMMCs (B). **A**: OTU set for 2010_GM_PC is colored bright green, 2010_GM_SE deep green, 2011_GM_SE yellow, 2012_GM_PC cyan, and 2012_GM_PC deep blue. **B**: total OTU set for 2010 GMMC is colored light green, 2011 GMMC yellow, and 2012 GMMC pale blue.

The above analyses comprehensively showed that a substantive fraction of GM_SE constituents were spatiotemporally constant while others were transitory in nature. However, the key questions that remained unresolved were the potential source(s) of so many newcomers into the GM_SE and the eventual fate of the incumbent populations that were apparently marginalized in course of community restructuring. Answers to these questions required a thorough idea about all plausible paths of dispersion and dispersal of populations as well as influx and efflux of bacteria within the entire GMMC continuum, and not just the GM_SE alone. This could be achieved only via a holistic comparison of the taxonomic diversity of the all GM_SEs and GM_PCs encountered in the PT microbiome over the three assessment years. Thus, V3 regions of all bacterial 16S rDNAs present in the two GM_PC samples were PCR-amplified and sequenced by Ion PGM up to depths ensuring plateaus in the rarefaction curves. The resultant OTU sets were then compared with the existing GM_SE OTU sets to generate comprehensive pictures of phylum (Fig. 7) and genus (Fig. 8) level diversities of the three GMMCs as a whole. Figure 8 additionally retraced the putative paths via which genus-level entities could have transmitted from one PT niche to another. The quotient of all demographic interrelations between pertinent mat sample pairs was determined by identifying their shared and unique OTUs (Fig. 9A). This was done by first merging the consensus sequences of all the OTUs of the two samples in question and then doing tree-based clustering at 97% similarity level. The doubletons of the new consensus sequence superset represented the OTUs common to the two samples. Similarly, final quotient of year on year addition, deduction and transmission of OTUs at the level of the whole GMMC was also determined (Fig. 9B). First, following the above principles a consensus sequence superset was created for 2010 or 2012 by taking all OTUs from the GM_PC and GM_SE samples of that year. The resultant superset, representing the total diversity of the 2010 or 2012 GMMC, was then merged with the consensus sequence set of 2011_GM_SE, and the clustering process repeated as above to get the common and unique OTUs between the GMMCs of 2010 and 2011, or 2011 and 2012 respectively.

Figure 8 revealed that a number of genera were dispersed throughout the GMMC continuum (see light green-highlighted names which are common to all PC or SE mat samples in question), while others were restricted to either PC or SE microenvironments (names highlighted in crimson, orange or light blue). While Fig. 8 identified only a few genera as common to all five mat samples, Fig. 9A indicated that the total count of such consistent broad niche-width OTUs could be quite high. Holistic comparison of the five OTU sets also offered a plausible reason for the sharp hike in the relative abundance of *Deinococcus-Thermus*, *Chloroflexi*, *Verrucomicrobia*, *Acidobacteria* or *Firmicutes* in the GM_SE edition of 2011 as against its 2010 counterpart. Since the 2010 PC niche was significantly rich in members of these phyla (see Fig. 7) it was natural to conjecture that between 2010 and 2011 spatiotemporal dispersal had occurred from the PC to the SE, thereby boosting the species richness and/or abundance of such bacteria in 2011_GM_SE. Fig 9A reinforced this notion by revealing that the total count of such niche-transcending OTUs (represented by the overlap between 2010_GM_PC and 2011_GM_SE) could be as high as 397, while the total number of native GM_SE OTUs conserved over 2010 to 2011 was 464. Concurrent to this, Fig. 8 identified seven such genera in 2011_GM_SE (names highlighted in deep blue) which were not present in the 2010_GM_SE but apparently migrated to the former from 2010_GM_PC. Subsequently, it was further interesting to detect all these seven genera in 2012_GM_PC while only two of them were archived in 2012_GM_SE. This gave the inkling that a sizeable fraction of the diversity accumulated in 2011_GM_SE from the previous year’s GM_PC actually migrated back to its original PC niche once that window of opportunity revived in 2012. The last notion was buttressed by the observation that in Fig. 9A, 562 out of the total 1248 OTUs of 2011_GM_SE overlapped with 2012_GM_PC, whereas only 438 OTUs of 2011_GM_SE overlapped with 2012_GM_SE. Corroboratively, the final quotient of diversity conservation in the PC niche over 2010–2012 was also as high as 515 (Fig. 9A).

Bacterial community structure of the mat-adjacent sediments was further studied to check whether the hypothesis of long-term survival and dispersal of the GMMC constituents was indeed true and the 2011 or 2012 mats were not constructed by similar founder bacteria from anywhere other than the GMMC. 2011_GM_SE-adjacent sediments were sampled thrice by scooping out 2.5 cm^3^ of freshly-precipitated mineral deposits in each round from three discrete points around, and at least 10 cm beyond, the mat structure. The three sub-samples were mixed thoroughly and labeled as the 2011_sediment sample. In 2012, sediments around the GM_SE and GM_PC growths were sampled separately as above; subsequently the six sub-samples were mixed thoroughly and treated as the 2012_sediment sample. Total community DNA was extracted from these two composite sediment samples and V3 regions of all bacterial 16S rDNAs present therein PCR-amplified and sequenced by Ion PGM up to depths that yielded plateaus in rarefaction curves. Table S11 documents the numerical details pertaining to the clustering of OTUs from the PGM reads of the 2011 and 2012 sediment samples.

**Figure 10:**
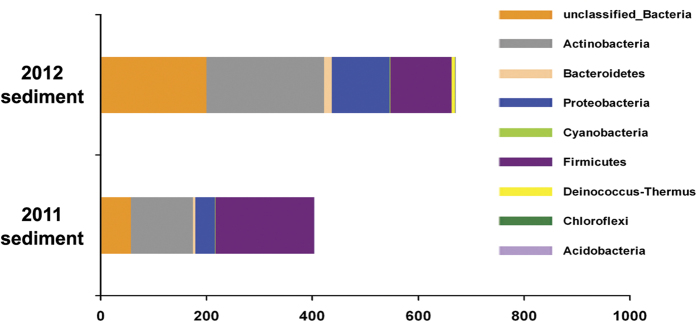
Phylum-level distribution of the bacterial OTUs identified in the mat-adjacent sediments. Note that in the 2011 and 2012 sediment samples number of OTUs belonging to *Cyanobacteria* (1 and 2 respectively)*, Deinococcus-Thermus* (0 and 6 respectively)*, Chloroflexi* (0 and 1 respectively) and *Acidobacteria* (1 and 1 respectively) were very low.

Taxonomic composition of the two sediment samples was quite similar to each other and at the same time clearly distinct from any of the five green mat samples. Bulk of their OTUs belonged to the phyla *Actinobacteria* and *Firmicutes* (Fig. 10), which, notably, had contributed very little to the alpha diversity of the GMMC samples (see Fig. 7). Most importantly, the 2011_sediment sample was found to include only one (viz. *Limnobacter*) out of the 20 genera hypothesized to have been handed over to 2011_GM_SE from 2010 GM_PC and/or GM_SE (compare the sedimentary genera listed in Table S12 with data shown in Fig. 8). Similarly, the 2012_sediment community included only four (viz. *Limnobacter*, *Meiothermus*, *Brevundimonas* and *Chloroflexus*) out of the 28 genera hypothesized to have been carried over to 2012 GM_PC and/or GM_SE from 2011_GM_SE (compare information given in Table S12 and Fig. 8). These observations, along with the fact that there was no microbial mat in the southern slope in 2010, reinforced the hypothesis that long-term survival and dispersal of 2010 GMMC constituents, and not the founder effect of similar bacteria from other mat-adjacent niches, created the framework of the 2011 and 2012 mats.

Figure 9A showed 679 OTUs to be common between 2010 and 2012 GM_SEs, thereby insinuating extensive species conservation within the GM_SE itself. Intriguingly, this number was much higher than the number of OTUs common between 2010 and 2011 GM_SE (464), or 2011 and 2012 GM_SE (438). These numbers were indicative of a scenario where several species native to the SE niche were pushed below detectable levels in 2011_GM_SE as this minimally-dispersed community was overcrowded by the influx of immigrants from discrete ecological niches, potentially extending beyond the GMMC framework. Then in 2012, when a large number of species apparently vacated the GM_SE space and migrated back to the revived PC niche, some of the GM_SE components marginalized in 2011 restored their populations above detectable limits. Similar results were also observed for the two GM_PCs, where the number of 2010 OTUs recovered from the 2012 edition was higher than the number of 2010_GM_PC OTUs which could have been transmitted via 2011_GM_SE. As such, in Fig. 9A, 515 OTUs were common to the 2010 and 2012 GM_PCs, while only 397 were common to 2010_GM_PC and 2011_GM_SE. These observations were further mirrored in Fig. 8 where some of the 2010_GM_PC and 2010_GM_SE genera that had gone undetected in 2011_GM_SE resurfaced in the 2012 GM_PC and/or GM_SE samples (see the hypothetical migration paths in dotted lines in Fig. 8). This suggested that within 2011_GM_SE, members coming from both 2010_GM_SE and 2010_GM_PC were equally starved for space and pushed below detectable levels.

The crux of the above analyses showed that a considerable portion of the 2011_GM_SE diversity was actually derived from 2010_GM_PC. At the same time, the apparent decline in the taxonomic diversity of the SE green mats in 2012 (or for that matter decline in the relative abundance of *Chloroflexi*, *Deinococcus-Thermus* and *Acidobacteria* in 2012_GM_SE) could be attributable to the partitioning of a substantial portion of 2011_GM_SE diversity into two discrete meta-communities. Since 2012_GM_PC was significantly richer than 2011_GM_SE in terms of the species diversity of *Chloroflexi*, *Deinococcus-Thermus* or *Acidobacteria* (see Fig. 7) it was natural to presume that between 2011 and 2012 bulk of these bacteria relocated to their preferred PC niches. In a nutshell, potential species loss notwithstanding, the overall diversity of the PT GMMC, over the three years, actually augmented from the 2010 level. The final quotient of the entire diversity dynamics of the PT GMMC is summarized below.

In 2010, the GMMC harbored a sum total of 2078 OTUs, while in 2012 the number went up to 2636 (Fig. 9B). These numbers, in conjunction with the data presented in Fig. 7, indicated a net increase in the alpha diversity of the GMMC, both in terms of OTU count and taxonomic spread of OTUs. Notably, the number of OTUs (618) common between 2010 and 2012 GMMC exceeded the overlaps between the GMMCs of 2010 and 2011 (409 OTUs) as well as 2011 and 2012 (451 OTUs). On the other hand, when the entire OTU set of the 2010 GMMC was compared with that of 2011_GM_SE, 1669 OTUs were unique to the former and only 839 were exclusive to the latter. The 839 OTUs detected exclusively in the 2011_GM_SE were most likely to represent foreign recruits that were not present in the PT GMMC earlier. While some idea about the generic identity of these OTUs can be made from the 32 names highlighted in yellow in Fig. 8, it is not impossible that some of these 839 were also present in the GMMC in 2010 but were overwhelmed by the preponderant *Proteobacteria*. Conversely, there were several pointers demonstrating that a substantive fraction of the 1669 OTUs found to be unique to the 2010 GMMC (versus 2011_GM_SE) were not totally lost from the 2011 community but marginalized below detection limits.

Remarkably again, in Fig. 9B, the number of OTUs common to 2011_GM_SE and the whole GMMC of 2012 (451) was higher than the overlap between 2010 GMMC and 2011_GM_SE (409 OTUs). This implied that many new recruits of 2011 were retained in the GMMC through 2012, a fact that concurred with the genus-level comparisons (note that in Fig. 8, 11 out of the 32 unique genera of 2011_GM_SE were also detected in 2012 GM_PC and/or GM_SE). Comparison between 2011_GM_SE and the 2012 GMMC further showed that 797 OTUs may have been lost in transition from 2011 to 2012, while 2185 OTUs apparently joined the GMMC during that period (idea about the generic identity of these OTUs can be made from the names highlighted by bright green, white and purple in Fig. 8). However, the net influx between 2010 - 2012 could have actually been lower than 2185, since this number also included those 2010 OTUs which were marginalized in 2011 (note that in Fig. 9B only 2018 OTUs were unique to the 2012 GMMC in comparison to 2010 GMMC). Most importantly, net diversity addition in the GMMC over three assessment years was surely higher than the net loss represented by the 1460 OTUs unique to the 2010 GMMC in comparison to the 2012 GMMC.

## Conclusions

GMMCs have been explored extensively from diverse high temperature habitats using multiple methodologies^26^,^27^,^28^,^29^,^30^. However, this study is distinctive in using large volume of metagenomic sequence in combination with an equally substantive amount of 16S rDNA sequence to unearth microbial diversity associated with a relatively uncommon type of circum-neutral hot spring system. Though globally infrequent, such hot springs, which are typically poor in TDS, silicate and chloride, but often rich in sodium/calcium and various compounds of sulfur, are common in the geothermal areas of North-Western Himalayas and Tibet^8^,^31^,^32^,^33^. But very few explorations have hitherto been conducted to characterize their microbiota^34^,^35^. However, the main importance of the present study was its elucidation of the survival dynamics of a GMMC in the face of drastic environmental changes. In conclusion, that survival story can be summed up as follows.

In 2010 a graded continuum of intricate ecological conditions existed along large swathes of the western slope of the PT sinter accretion. This resulted in wide habitat heterogeneity within the ecocline comprised of several gradients of opportunity (mainly in the form of various nutrients dissolved in the hot water outflow) as well as resistance (mainly in the form of thermal stress). While some of the opportunity gradients lay parallel to the gradient of environmental resistance, others were aligned antiparallel to the latter. Since almost none of the GMMC constituents were hyperthermophilic, the temperature gradient, running from the proximal channels to the slope environment, acted as a potential gradient of environmental resistance for nearly all the members of the community. In this scenario, ecological growth of the GMMC in time and space was mainly driven by the dispersion of populations, which in turn depended on the innate ability (resilience) of the species to cope with the environmental resistance. Eventually, on the multivariate ecological continuum (or the ecocline) the diverse biotypes (taxonomic and/or metabolic types) potentially consolidated their positions at their respective optimum niches, while their populations tapered off gradually on either side of the consolidation zones.

Then between November 2010 and 2011 the large geyser atop the PT got extinguished, thereby causing the hot water flow on western slope of the mound to dry up completely. Even as substratum desiccation turned the existing habitat of the GMMC hostile, during the same period a slender prospect of survival (habitable space) opened up in the form of two new vents in the southern slope of the PT. Amidst this spatial drift, as well as shrinkage, of habitable space (defined by the shorter length and breadth of the new OFC) dispersal emerged as the main driver of ecological sustenance of the community. While some incumbent populations of the sprawling 2010 GMMC compromised their niche specialization (e.g. *Deinococcus-Thermus*) others gave up their dispersion (e.g. *Proteobacteria*) to launch spatiotemporal dispersal and build a minimally-dispersed seed-like community (viz. the 2011_GM_SE) on the southern-slope OFC. The apparent aim of the whole process was to conserve maximum-possible phylogenomic diversity from both ends of the 2010 GMMC continuum. However, in course of this en masse dispersal several incumbent populations of the 2010 GMMC got eliminated or acutely marginalized, even as many of them managed to maintain significant representation in the GM_SE construct of 2011. Besides embracing internally-displaced populations from the 2010 GMMC, 2011_GM_SE showed exceptional receptivity in countenancing external immigrants. This openness potentially enhanced the ecological fitness (survival value) of the community by inducting new metabolic pathways and/or variants of existing ones. Presumably, 2011_GM_SE must have positioned itself at such an optimum point on the new ecocline that allowed its diverse constituents to make best use of all available environmental opportunities and overcome existing resistances. Growth (dispersive scope) of 2011_GM_SE, however, was constrained by additional environmental resistances such as limited habitable space and scarce nutrients due to diminutive volume, flow rate, and chemical-content of the hydrothermal discharge. Presumably in order to sustain the equilibrium of its existence this recuperating GMMC limited its dispersion to a restricted zone of the already short ecological continuum, and therefrom started developing resilience adequate enough to encounter the existing resistances and prepare for future growth.

Finally in 2012, geothermal activity in the southern-slope of the PT gained impetus (in the form of new vents, and increased flow rates and mineral contents of discharges), thereby widening the available ecological niches. The eco-physiologically miscellaneous components of 2011_GM_SE took this opportunity to split into discrete GM_PC and GM_SE metacommunities. As many expatriated 2010_GM_PC populations vacated the SE refuge, community growth space in the SE niche increased further. Thus, many GM_SE natives (e.g. many *Proteobacteria*) who were reduced to minuscule minorities in 2011_GM_SE regained preeminence in 2012_GM_SE. However, the second round of population dispersal was more complex than the 2010–2011 transition since many more new species joined the GMMC, especially through the revived GM_PC window. In this way the GMMC seeds were again successfully sown at either end of the thermo-nutritional gradient. Whether this niche-partitioned GMMC can regain dispersive growth and regenerate an expansive continuum depend on the vitality of the spring system.

## Methods

### Sampling and *in situ* analyses

Samples were collected in sterile polypropylene bottles and transported immediately to the laboratory in insulated 4 °C coolers. At all the five data points sampling was done thrice by scraping off 2.5 cm^2^ of mat material in each round from three horizontally discrete points (5 to 10 cm apart) equidistant from the water source and having identical temperature and pH. Utmost care was taken to ensure that the green mat structures were exclusively scraped off from the surface with minimum infiltration of underlying sediments. Immediately upon reaching the laboratory, all sub-samples of a given mat sample were mixed thoroughly by rehydrating with sterile 0.9% NaCl solution. Total community DNA (metagenome), in quantities sufficient for all downstream experiments, was extracted from these composite samples using PowerMax soil DNA isolation kit (MoBio).

S_2_O_3_^2−^ and SO_4_^2−^ concentrations in the thermal fluid were measured *in situ* by iodometric titration and gravimetric precipitation (as BaSO_4_) respectively^36^,^37^,^38^,^39^,^40^. S^2−^ in the thermal waters was measured using the chemical principle that N, N-dimethyl-p-phenylene diamine dihydrochloride combines quantitatively with H_2_S in the presence of FeCl_3_ catalyst and HCl to give a blue colored complex. Fe^3+^ concentration in the waters was determined by thiocyanate colorimetry. Quantitative spectrophotometric determination of Fe^2+^ was done by reacting the same with o-phenanthroline *in situ* and eliminating the interference of Fe^3+^ by complexing it with sodium fluoride^41^. Details of analytical methods are provided as Supplementary Information.

### Shotgun metagenomics

Metagenomes were shotgun sequenced by the Ion Torrent Personal Genome Machine (Ion PGM) or the Ion Proton platform^42^ (Life Technologies) using 200 bp read chemistry on the Ion 318 or the PI V2 chip respectively (details in Supplementary Information). The metagenomic sequence sets were quality-filtered, and clipped to eliminate low quality regions, using sff_extract. The sequence files were deposited to the NCBI Sequence Read Archive with the run accession numbers SRR1662204, SRR1662223 and SRR1662248 under the BioProject accession number PRJNA268268.

Read sets were annotated and analyzed using MG-RAST^43^ to gain quantitative insights into the community structures and functions. Within this analysis pipeline, sequences were trimmed so as to contain no more than five consecutive bases below the phred score of 15. Reads were taxonomically classified using the “Organism Abundance” tool of MG-RAST following either the “Best Hit Classification” (BHC) or the “Lowest Common Ancestor” (LCA) approach. For higher-level classifications (up to class) shotgun metagenomic read sets were searched by blastx against the non-redundant (*nr*) protein database following the BHC approach with minimum alignment length of 45 bp (15 amino acids) and minimum identity cutoff of 60%. Alternatively, to classify reads up to the genus level similar blastx searches were conducted using the LCA algorithm with minimum alignment length of 30 amino acids and minimum identity cutoff of 70%. Each read set was also searched for small subunit (*ssu*) rRNA genes against the RDP database using blastn with minimum alignment length of 50 bp and 70% minimum identity cutoff. Sequences were potentially assigned to genera only when they shared >95% rRNA sequence identity with known species. Maximum e-value cutoff used in all these analyses was 1e-5.

Presence of only those genera which accounted for ≥0.01% of all classifiable metagenomic reads was considered to be significant within any given community. This convention led to the short-listing of around 300 (out of a total 600), 500 (out of a total 1700), and 400 (out of a total 1900) genus-level entities from the ‘organism abundance’ list of the 2010, 2011 and 2012 GM_SE metagenomes respectively. Genera falling below this cutoff were thought to be present in the community in very low proportions and thus kept out of all ecological interpretations.

Ecological diversity was quantified from shotgun metagenomic data by rendering the phylum-level distribution of reads (a measure of relative abundance of phyla) to Simpson Dominance and, Shannon–Wiener Diversity and Evenness indices^44^ (detailed formulae are given in Supplementary Information).

### Ordination

Several correlation and ordination methods were used to identify potential bacterial groups that had added importance in the present GMMC dynamics. Pearson (linear) correlations between phyla/class-level metagenomic data and physicochemical properties of the hydrothermal fluid were calculated by XLSTAT (http://www.xlstat.com/en/download.html). PCoA and CCA were carried out in STATISTICA 10.0 for Windows (http://statistica.software.informer.com/10.0/) and validated using PAST 3.02^45^. PAST was also used to determine Euclidean distance and Bray-Curtis similarity between the GM_SE samples.

### Species richness estimation (OTU clustering)

Amplification of V3 regions of 16S rRNA genes and sequencing of amplicon pools by Ion PGM were carried out using fusion primers^46^ (details in Supplementary Information). The sequence files for the GMMC samples were deposited to the NCBI Sequence Read Archive (SRA) with the run accession numbers SRR1663456, SRR1663458, SRR1663464, SRR1663465 and SRR1663466 under the BioProject accession number PRJNA268268. The sequence files for the two mat-adjacent sediment samples were deposited to the SRA with the run accession numbers SRR1800757 and SRR1802680 under the BioProject accession number PRJNA268268.

Raw V3 region-specific reads were first filtered for high quality value (QV 20) and length threshold of 100 bp^46^. Selected reads were then converted to fasta from fastq using Fastx_toolkit (v0.0.13.2). Subsequent preprocessing using the “preproc” module of ESPRIT^47^ involved screening of only those reads which had intact 16S rDNA universal primer sequence plus the barcode adaptor and the barcode. Adaptor sequences were then trimmed; reads containing unidentified bases (N) filtered out together with those which were identical to another read or were mere subsets of a longer read. After preprocessing, denoising of reads was achieved as follows: (i) pairwise alignment of reads by Needleman algorithm was carried out using the module “pairwise.seqs” from MOTHUR^48^; (ii) the alignment matrix thus created was utilized for Single Linkage Preclustering^49^ using a script^50^ provided by the VAMPS (Visualization and Analysis of Microbial Population Structure project) integrated collection of tools located at http://vamps.mbl.edu/overview.php. Pairwise distance of 0.02 (98% identity) was used as threshold criterion for selecting reads in preclustering, which were then stored in fasta format.

Reads were clustered by hierarchical clustering technique using various modules available within ESPRIT^47^. In brief, reads were again aligned pairwise following Needleman algorithm; clusters or operational taxonomic units (OTUs) were created with pairwise distance of 0.03 (97% identity) as the criterion; and eventually the total number of OTUs identified in a community gave the estimate of its species richness. The clusters or OTUs thus formed were also used to draw statistical inferences. OTUs were filtered using a Perl script to remove all the singletons. Singletons were removed from *.Cluster as well as *.Cluster_List to make new_Cluster & new_Cluster_List files. The new Cluster files were run in the statistical module of ESPRIT to get ACE and Rarefaction analyses. Rarefaction data were further utilized in R package^51^ to create graphs for reads taking part in OTU formation versus number of OTUs formed. Consensus sequences were created using the clusters (minus singletons), and the fasta and frequency files created during the clustering operations. All consensus sequences generated for a given dataset were taxonomically classified with the help of the “RDP Classifier” located at http://rdp.cme.msu.edu/classifier/classifier.jsp.

## Additional Information

**How to cite this article**: Ghosh, W. *et al.* Resilience and receptivity worked in tandem to sustain a geothermal mat community amidst erratic environmental conditions. *Sci. Rep.* **5**, 12179; doi: 10.1038/srep12179 (2015).

## Supplementary Material

Supplementary Information

## Figures and Tables

**Table 1:** Physicochemical properties of the hot water flowing over the PC as well as SE green mats on the day of their sampling.

	2010		2011	2012	
	GM_PC	GM_SE	GM_SE	GM_PC	GM_SE
Temperature (°C)	75	65	60	75	65
pH	7.5	7.25	6.75	7.75	7.0
TDS (g l^−1^)	1	0.7	0.3	0.8	0.5
Sulfate content (mM)	2.5	2.1	3.2	3	2.5
Sulfide content (μM)	232	290	170	155	203
Thiosulfate content (mM)	5	4.2	2.6	4.5	3.7
Ferric (μM)	50	20	10	40	20
Ferrous (mM)	6.0	4.8	1.2	5.0	2.5
Acetate (μM)	15	12	5	15	15
Formate (μM)	20	15	5	15	10

Given values are averages of three different experiments performed at six hour intervals over the day. Each experiment in its turn involved three assay replicates. Standard deviation for none of the values was >2%.

**Table 2:** OTU counts and other derived values utilized in *T*_*R*_ calculation for the three GM_SE communities.

Name of the phylum	Hypothetical superset for the GM_SE system				2010_GM_SE				2011_GM_SE				2012_GM_SE			
	No. of OTUs possible in the GM_SE system	p_i_	√n_i_	(p_i_) (√n_i_)	No. of OTUs in the GM_SE edition (n_i_)	p_i_ = (n_i_/N_i_)	√n_i_	(p_i_) (√n_i_)	No. of OTUs in the GM_SE edition (n_i_)	p_i_ = (n_i_/N_i_)	√n_i_	(p_i_) (√n_i_)	No. of OTUs in the GM_SE edition (n_i_)	p_i_ = (n_i_/N_i_)	√n_i_	(p_i_) (√n_i_)
*Actinobacteria*	8	1	2.8284271	2.83	1	0.13	1	0.13	6	0.75	2.44948974	1.84	1	0.13	1	0.13
*Spirochaetes*	7	1	2.6457513	2.65	2	0.29	1.414213562	0.41	6	0.86	2.44948974	2.11	3	0.43	1.732051	0.74
*Bacteroidetes*	387	1	19.672316	19.67	207	0.54	14.38749457	7.77	206	0.53	14.3527001	7.61	85	0.22	9.219544	2.03
*Proteobacteria*	1429	1	37.802116	37.8	768	0.54	27.71281292	14.96	492	0.35	22.181073	7.76	716	0.5	26.75818	13.38
*Cyanobacteria*	24	1	4.8989795	4.9	24	1	4.898979486	4.9	1	0.04	1	0.04	6	0.25	2.44949	0.61
*Nitrospira*	18	1	4.2426407	4.24	1	0.06	1	0.06	18	1	4.24264069	4.24	0	0	0	0
*Acidobacteria*	11	1	3.3166248	3.32	1	0.09	1	0.09	10	0.91	3.16227766	2.88	2	0.18	1.414214	0.25
*Aquificae*	2	1	1.4142136	1.41	1	0.5	1	0.5	1	0.5	1	0.5	0	0	0	0
*Deinococcus-Thermus*	154	1	12.409674	12.41	6	0.04	2.449489743	0.1	154	1	12.4096736	12.41	5	0.03	2.236068	0.07
*Firmicutes*	7	1	2.6457513	2.65	0	0	0	0	3	0.43	1.73205081	0.74	5	0.71	2.236068	1.59
*Chloroflexi*	8	1	2.8284271	2.83	0	0	0	0	7	0.88	2.64575131	2.33	1	0.13	1	0.13
*Armatimonadetes*	2	1	1.4142136	1.41	0	0	0	0	1	0.5	1	0.5	1	0.5	1	0.5
*Verrucomicrobia*	3	1	1.7320508	1.73	0	0	0	0	3	1	1.73205081	1.73	1	0.33	1	0.33
TM7	1	1	1	1	0	0	0	0	1	1	1	1	0	0	0	0
WS3	1	1	1	1	0	0	0	0	1	1	1	1	0	0	0	0
*Gemmatimonadetes*	1	1	1	1	0	0	0	0	1	1	1	1	0	0	0	0
*Unclassified Bacteria*	944	1	1	1	467	0.49	21.61018278	10.59	337	0.36	18.3575598	6.61	394	0.42	19.84943	8.34
Sum total (Σ)	3007*			101.85	1478			39.51	1248			54.3	1220			28.1

^*^Out of these 3007 OTUs there were 2164 singletons (representing those species which were found only once in the SE green mats), 747 doubleton (representing species which were common to any two of the three SE green mats) and 96 tripletons, which represented only those OTUs which were common to all three GM_SE editions. These 96 most-conserved OTUs, again, were distributed over *Proteobacteria* (55), *Bacteroidetes* (11) and *Cyanobacteria* (03), while the 27 rest were Unclassified Bacteria.

## References

[b1] Wèachtershèauser G. in Origin of life in an iron-sulfur world, 206–218 (Cambridge University Press, Cambridge; New York, 1998).

[b2] Martin W., Baross J., Kelley D. & Russell M. J. Hydrothermal vents and the origin of life. Nat. Rev. Microbiol. 6, 805–814 (2008). 18820700 10.1038/nrmicro1991

[b3] Martin W. & Russell M. J. On the origin of biochemistry at an alkaline hydrothermal vent. Phil. Trans. R. Soc. B. 362, 1887–1925 (2007). 17255002 10.1098/rstb.2006.1881 PMC2442388

[b4] Farmer J. D. Hydrothermal Systems: Doorways to Early Biosphere Evolution. GSA Today 10, 1–9 (2000).

[b5] Hessler R. R. *et al.* Temporal change in megafauna at the Rose Garden hydrothermal vent (Galapagos Rift; eastern tropical Pacific). Deep Sea Research Part A. Oceanographic Research Papers. 35, 1681–1709 (1988).

[b6] Johnson K. S., Childress J. J., Hessler R. R., Sakamoto-Arnold C. M. & Beehler C. L. Chemical and biological interactions in the Rose Garden hydrothermal vent field, Galapagos spreading center. Deep Sea Research Part A. Oceanographic Research Papers 35, 1723–1744 (1988).

[b7] Shank T. M. *et al.* Time-Series Exploration and Biological, Geological, and Geochemical Characterization of the Rosebud and Calyfield Hydrothermal Vent Fields at 86°W and 89.5°W on the Galapagos Rift in *Eos Trans*. American Geophysical Union, 83(47), Fall Meet. Suppl., Abstract T11C-1257. San Francisco, CA: AGU. (2002, December 6-10).

[b8] Rai A. P. Compilation of data on chemical analysis of water and gas samples from North West Himalaya and adjoining areas, (Geological Survey of India, Kolkata, 2001).

[b9] Overmann J. & Garcia-pichel F. The Phototrophic Way of Life in The Prokaryotes. Vol. 2 (eds Dworkin M. *et al.* ). 32–85 (Springer, New York, 2006).

[b10] Ward N. L. *et al.* Three genomes from the phylum Acidobacteria provide insight into the lifestyles of these microorganisms in soils. Appl. Environ. Microbiol. 75, 2046–56 (2009). 19201974 10.1128/AEM.02294-08 PMC2663196

[b11] Johnson D. B., Bacelar-Nicolau P., Okibe N., Thomas A. & Hallberg K. B. Ferrimicrobium acidiphilum gen. nov., sp. nov. and Ferrithrix thermotolerans gen. nov., sp. nov.: heterotrophic, iron-oxidizing, extremely acidophilic actinobacteria. Int. J. Syst. Evol. Microbiol. 59, 1082–1089 (2009). 19406797 10.1099/ijs.0.65409-0

[b12] Ghosh W. & Dam B. Biochemistry and molecular biology of lithotrophic sulfur oxidation by taxonomically and ecologically diverse bacteria and archaea. FEMS Microbiol. Rev. 33, 999–1043 (2009). 19645821 10.1111/j.1574-6976.2009.00187.x

[b13] Hanada S. & Pierson B. K. The Family Chloroflexaceae in The Prokaryotes, Vol. 7, (eds Dworkin M. *et al.* ) 815–842 (Springer, New York, 2006).

[b14] Schwartz E. & Friedrich B. The H_2_-Metabolizing Prokaryotes in The Prokaryotes. Vol. 2, (eds Dworkin M. *et al.*) 496–563 (Springer, New York, 2006).

[b15] Kim S. B., Falconer C., Williams E. & Goodfellow M. Streptomyces thermocarboxydovorans sp. nov. and Streptomyces thermocarboxydus sp. nov., two moderately thermophilic carboxydotrophic species from soil. Int. J. Syst. Bacteriol. 48, 59–68 (1998). 9542077 10.1099/00207713-48-1-59

[b16] Kim S. B. & Goodfellow M. Streptomyces thermospinisporus sp. nov., a moderately thermophilic carboxydotrophic streptomycete isolated from soil. Int. J. Syst. Evol. Microbiol. 52, 1225–8 (2002). 12148632 10.1099/00207713-52-4-1225

[b17] Emerson D., Fleming E. J. & McBeth J. M. Iron-oxidizing bacteria: an environmental and genomic perspective. Annu. Rev. Microbiol. 64, 561–583 (2010). 20565252 10.1146/annurev.micro.112408.134208

[b18] Heidelberg J. F. *et al.* Genome sequence of the dissimilatory metal ion-reducing bacterium Shewanella oneidensis. Nat. Biotechnol. 20, 1118–1123 (2002). 12368813 10.1038/nbt749

[b19] Drake H. L., Küsel K. & Matthies C. Acetogenic Prokaryotes in The Prokaryotes, Vol. 2, (eds Dworkin M. *et al.* ) 354–420 (Springer, New York, 2006).

[b20] Whitman W. B., Bowen T. I. & Boone D. R. The Methanogenic Bacteria in The Prokaryotes, Vol. 3, (eds Dworkin M. *et al.* ) 165–207 (Springer, New York, 2006).

[b21] Holtmann G. & Bremer E. Thermoprotection of Bacillus subtilis by exogenously provided glycine betaine and structurally related compatible solutes: involvement of Opu transporters. J. Bacteriol. 186, 1683–93 (2004). 14996799 10.1128/JB.186.6.1683-1693.2004 PMC355977

[b22] Nau-Wagner G. *et al.* Genetic control of osmoadaptive glycine betaine synthesis in Bacillus subtilis through the choline-sensing and glycine betaine-responsive GbsR repressor. J. Bacteriol. 194, 2703–14 (2012). 22408163 10.1128/JB.06642-11 PMC3347207

[b23] Imhoff J. F. The Phototrophic Alpha-Proteobacteria in The Prokaryotes. Vol. 5, (eds Dworkin M. *et al.* ) 41–64 (Springer, New York, 2006).

[b24] Bowman J. P. The Methanotrophs—The Families Methylococcaceae and Methylocystaceae in The Prokaryotes, Vol. 5, (eds Dworkin M. *et al.* ) 266–289 (Springer, New York, 2006).

[b25] Lieberman R. L. & Rosenzweig A. C. Crystal structure of a membrane-bound metalloenzyme that catalyses the biological oxidation of methane. Nature 434, 177–182 (2005). 15674245 10.1038/nature03311

[b26] Yamamoto H. *et al.* Phylogenetic Evidence for the Existence of Novel Thermophilic Bacteria in Hot Spring Sulfur-Turf Microbial Mats in Japan. Appl. Environ. Microbiol. 64, 1680–1687 (1998). 9572936 10.1128/aem.64.5.1680-1687.1998 PMC106215

[b27] Takacs C. D. *et al.* Phylogenetic characterization of the blue filamentous bacterial community from an Icelandic geothermal spring. FEMS Microbiol. Ecol. 35, 123–128 (2001). 11295450 10.1111/j.1574-6941.2001.tb00795.x

[b28] Hiraishi A., Umezawa T., Yamamoto H., Kato K. & Maki Y. Changes in Quinone Profiles of Hot Spring Microbial Mats with a Thermal Gradient. Appl. Environ. Microbiol. 65, 198–205 (1999). 9872780 10.1128/aem.65.1.198-205.1999 PMC91003

[b29] Reysenbach A. L., Wickham G. S. & Pace N. R. Phylogenetic analysis of the hyperthermophilic pink filament community in Octopus Spring, Yellowstone National Park. Appl. Environ. Microbiol. 60, 2113–9 (1994). 7518219 10.1128/aem.60.6.2113-2119.1994 PMC201609

[b30] Skirnisdottir S. *et al.* Influence of sulfide and temperature on species composition and community structure of hot spring microbial mats. Appl. Environ. Microbiol. 66, 2835–41 (2000). 10877776 10.1128/aem.66.7.2835-2841.2000 PMC92081

[b31] Ghosh W. *et al.* Molecular and Cellular Fossils of a Mat-Like Microbial Community in Geothermal Boratic Sinters. Geomicrobiology Journal 29, 879–885 (2012).

[b32] Shanker R. *et al.* Geothermal Atlas of India 144 (Geological Survey of India, Kolkata, 1991).

[b33] Thussu J. L. Geotherman Evergy Resources of Indian. (Geological Survey of India, Kolkata, 2002).

[b34] Huang Q. *et al.* Archaeal and bacterial diversity in hot springs on the Tibetan Plateau, China. Extremophiles 15, 549–63 (2011). 21695489 10.1007/s00792-011-0386-z

[b35] Song Z.Q. *et al.* Bacterial and archaeal diversities in Yunnan and Tibetan hot springs, China. Environ. Microbiol. 15, 1160–75 (2013). 23126508 10.1111/1462-2920.12025

[b36] Mazumdar A. & Strauss H. Sulfur and strontium isotopic compositions of carbonate and evaporite rocks from the late Neoproterozoic-early Cambrian Bilara Group (Nagaur-Ganganagar Basin, India): Constraints on intrabasinal correlation and global sulfur cycle. Precambrian Research 149, 217–230 (2006).

[b37] Dam B., Mandal S., Ghosh W., Das Gupta S. K. & Roy P. The S4-intermediate pathway for the oxidation of thiosulfate by the chemolithoautotroph Tetrathiobacter kashmirensis and inhibition of tetrathionate oxidation by sulfite. Res. Microbiol. 158, 330–8 (2007). 17509837 10.1016/j.resmic.2006.12.013

[b38] Ghosh W., Bagchi A., Mandal S., Dam B. & Roy P. Tetrathiobacter kashmirensis gen. nov., sp. nov., a novel mesophilic, neutrophilic, tetrathionate-oxidizing, facultatively chemolithotrophic betaproteobacterium isolated from soil from a temperate orchard in Jammu and Kashmir, India. Int. J. Syst. Evol. Microbiol. 55, 1779–87 (2005). 16166666 10.1099/ijs.0.63595-0

[b39] Kelly D. P. & Wood A. P. Synthesis and Determination of Thiosulfate and Polythionates. Methods in Enzymology 243, 475–501 (1994).

[b40] Alam M., Pyne P., Mazumdar A., Peketi A. & Ghosh W. Kinetic enrichment of 34S during proteobacterial thiosulfate oxidation and the conserved role of SoxB in S-S bond breaking. Appl. Environ. Microbiol. 79, 4455–64 (2013). 23686269 10.1128/AEM.00956-13 PMC3697525

[b41] Herrera L., Ruiz P., Aguillon J. C. & Fehrmann A. A new spectrophotometric method for the determination of ferrous iron in the presence of ferric iron. Journal of Chemical Technology & Biotechnology 44, 171–181 (1989).

[b42] Rothberg J. M. *et al.* An integrated semiconductor device enabling non-optical genome sequencing. Nature 475, 348–52 (2011). 21776081 10.1038/nature10242

[b43] Meyer F. *et al.* The metagenomics RAST server - a public resource for the automatic phylogenetic and functional analysis of metagenomes. BMC Bioinformatics 9, 386 (2008). 18803844 10.1186/1471-2105-9-386 PMC2563014

[b44] Magurran A. E. Measuring Biological Diversity, (Blackwell Publishing Limited, Oxford, UK, 2003).

[b45] Hammer Ø., Harper D. A. T. & Ryan P. D. PAST: Paleontological statistics software package for education and data analysis. Palaeontologia Electronica 4, 9 (2011).

[b46] Yergeau E. *et al.* Next-generation sequencing of microbial communities in the Athabasca River and its tributaries in relation to oil sands mining activities. Appl. Environ. Microbiol. 78, 7626–37 (2012). 22923391 10.1128/AEM.02036-12 PMC3485728

[b47] Sun Y. *et al.* ESPRIT: estimating species richness using large collections of 16S rRNA pyrosequences. Nucleic Acids Res. 37, e76 (2009). 19417062 10.1093/nar/gkp285 PMC2691849

[b48] Schloss P. D. *et al.* Introducing mothur: open-source, platform-independent, community-supported software for describing and comparing microbial communities. Appl. Environ. Microbiol. 75, 7537–41 (2009). 19801464 10.1128/AEM.01541-09 PMC2786419

[b49] Junemann S. *et al.* Bacterial community shift in treated periodontitis patients revealed by ion torrent 16S rRNA gene amplicon sequencing. PLoS One 7, e41606 (2012). 22870235 10.1371/journal.pone.0041606 PMC3411582

[b50] Huse S. M., Welch D. M., Morrison H. G. & Sogin M. L. Ironing out the wrinkles in the rare biosphere through improved OTU clustering. Environ. Microbiol. 12, 1889–98 (2010). 20236171 10.1111/j.1462-2920.2010.02193.x PMC2909393

[b51] Dixon P. VEGAN, a package of R functions for community ecology. Journal of Vegetation Science 14, 927–930 (2003).

