# Supplementary material for: Resilience and receptivity worked in tandem to sustain a geothermal mat community amidst erratic environmental conditions
Source: Sci Rep. 2015 Jul 17;5:12179. doi: 10.1038/srep12179 (PMC4505329; doi:10.1038/srep12179)
Supplement: Supplementary Information [file srep12179-s1.doc]

**Supplementary Information for the paper**

**Resilience and receptivity worked in tandem to sustain a geothermal mat community amidst erratic environmental conditions**

Wriddhiman Ghosh^1^*, Chayan Roy^1#^, Rimi Roy^1#^, Pravin Nilwe^2^, Ambarish Mukherjee^3^, Prabir Kumar Haldar^1^, Neeraj Kumar Chauhan^4^, Sabyasachi Bhattacharya^1^, Atima Agarwal^4^, Ashish George^4^, Prosenjit Pyne^1^, Subhrangshu Mandal^1^, Moidu Jameela Rameez^1^ and Goutam Bala^3^

1, Department of Microbiology, Bose Institute, P-1/12 CIT Scheme VIIM, Kolkata - 700054, India

2, Thermo Fisher Scientific, Vatika Business Centre, Supreme Business Park, Hiranandani Gardens, Mumbai 400076 India

3, Department of Botany, The University of Burdwan, Burdwan, West Bengal, PIN - 713104, India.

4, Thermo Fisher Scientific, 372 Udyog Vihar Phase II, Gurgaon, Haryana - 122016, India

# Equal contribution

**Methods**

***Analytical methods***

Thiosulfate and sulfate concentrations in the hydrothermal fluid were measured *in situ* by iodometric titration and gravimetric precipitation respectively(Kelly & Wood, 1994, Gho*sh, et a*l., 2005, D*am, et a*l., 2007, Al*am, et a*l., 2013).

The chemical principle for iodometric titration was that K_2_Cr_2_O_7_ reacts with KI in presence of HCl according to the reaction

6KI + 14HCl + K_2_Cr_2_O_7_ = 2CrCl_3_ + 3I_2_ + 7H_2_O + 8KCl

Now, if some starch solution is added to the above solution prepared in such a way as to contain a small amount of iodine, it forms a blue starch-iodine conjugate. Then, if Na_2_S_2_O_3_ solution is added to this solution, the blue color of the starch-iodine complex disappears as soon as all the iodine is reduced to iodide ion following the reaction

Starch-Iodine + 6 S_2_O_3_^2–^ = Starch + 6 I^–^ + 3 S_4_O_6_^2–^

(2 Na_2_S_2_O_3_ + I_2_ = Na_2_S_4_O_6_ + 2 NaI)

To set the reaction, initially 3.0 g KI was dissolved in a solution containing 10 ml of 0.01 N K_2_Cr_2_O_7_, 10 ml HCl and 49 ml H_2_O in a 1000 ml conical flask. After mixing the solution well, the flask was covered with a watch glass and then incubated in dark for 5-10 minutes. 230 ml of H_2_O and 1 ml of 1 % starch solution was then added and mixed. The solution became blue after the addition of starch. 1 ml of sample containing unknown quantity of thiosulfate was added and the mixture was then titrated with 0.01 N sodium thiosulfate until the solution became clear. Concentration of thiosulfate was then calculated by plotting burette readings onto standard curve prepared using thiosulfate solutions of known concentrations as samples.

To precipitate dissolved sulfate as BaSO_4_, 5 ml of hot water was acidified to pH 2.0 with dilute HCl, and heated at ~90 ^O^C for 20 minutes. Excess of preheated 0.05 M (0.1 N) BaCl_2_ solution was added to the acidified water, stirring vigorously all the while(Mazumdar & Strauss, 2006). The precipitates formed after addition of BaCl_2_ were digested for two hours at 90 ^O^C and decanted through fine-pore ash-less Whatman No. 42 filter paper. They were subsequently washed thrice by dilute HCl, re-filtered as above; and the moist filter papers folded into small cones and then allowed to dry for several days in uncovered crucibles. Finally upon reaching the laboratory, the dry white BaSO_4_ powders were recovered by burning away the filter papers, and then weighed for estimation of sulfate content.

Sulfide in the thermal waters was measured using the chemical principle that N, N-dimethyl-p-phenylene diamine dihydrochloride combines quantitatively with H_2_S in the presence of FeCl_3_ catalyst and HCl give a blue colored complex. The following reagents and standards were prepared in de-ionized oxygen free water/acid prepared by bubbling nitrogen gas through a suitable amount of water/acid for 30-60 minutes.

1. 0.2g of N, N-dimethyl-p-phenylene diamine dihydrochloride dissolved in 100 ml of ~6M oxygen-free hydrochloric acid.
2. 1.6g of ferric chloride (FeCl_3_) dissolved in 100 ml of ~6M oxygen-free hydrochloric acid.

To prepare calibration standards and blanks 2-2.5 g of Na_2_S was dissolved in 100 ml of oxygen-free water. 1ml of this stock solution was taken and diluted to 100 ml in a volumetric flask to prepare the working solution. From this, 0.5, 1, 1.5, 2 and 2.5 ml were pipetted out in five 100 ml volumetric flasks. 1 ml each of the reagents N, N-dimethyl-p-phenylene diamine dihydrochloride and ferric chloride (FeCl_3_) were added to each of the flasks and the volumes made up to 100 ml. These were the calibration standards. A blank was also prepared by adding 1 ml each of the reagents to 98 ml of deionized water.

For standardization of the sulfide stock solution 5 ml aliquots were taken from the stock solution and a suitable amount of silver nitrate (AgNO_3_) solution was added to these in such a way that the entire sulfide in the solution was converted to a black silver sulfide (Ag_2_S) precipitate. The solutions were filtered through pre-weighed 0.45 micron Millipore membrane filter papers. The filter papers were dried at 50 ^O^C and weighed with the precipitate after drying. From the weight of the dry Ag_2_S precipitate, we calculated the sulfide originally present in the stock solution. From molecular weight of Ag_2_S it was assumed that 247.8g of Ag_2_S contained 32.0 g sulfur. Hence, if the weight of precipitate obtained was 1 g then the stock solution originally contained (32*1)/247.8 = 0.1291g S. From this we could calculate the weight of sulfur in the calibration standards and obtain the concentration in µM of sulfide.

For calibration and measurement of samples 1 ml of each of the reagents was added to the samples precipitated from the thermal water *in situ* as Ag_2_S. For this purpose suitable dilutions were made so as to ensure that the resulting blue color was not too intense. The calibration standards and samples were measured in a spectrophotometer at 670 nm ~1 hour after the addition of reagents. The blank was measured first, followed by the standards in increasing order of concentrations. The absorptions were plotted against the concentrations and the (straight) calibration line was obtained. Absorptions of the samples were extrapolated on the calibration line and concentrations calculated accordingly.

Ferric iron (Fe^3+^) concentration in the thermal water was determined by thiocyanate colorimetry. Excess of thiocyanate (SCN^−^) was added to the sample so as to make all Fe^3+^ ions react to it forming the blood-red complex [FeSCN]^2+^. The reaction was performed individually on the samples as well as on a series of standards having known Fe^3+^ concentrations. Then by comparing the intensity of the color of the sample solution with that of the standards the concentration of Fe^3+^ in the sample was determined. Quantitative spectrophotometric determination of Fe^2+^ was done by reacting the same with o-phenanthroline *in situ* and eliminating the interference of Fe^3+^ by complexing it with sodium fluoride, as described elsewhere (Herre*ra, et a*l., 1989). Spectrophotometric readings were taken against a blank containing all reagents except the sample, which was replaced by distilled water. Absorbance was read at 510nm.

***Metagenome sequencing***

Quality of the DNAsamples was checked by electrophoresis and considered to be of high quality when no degradation signs were apparent. DNA quantity was determined using Qubit dsDNA HS Assay Kit (Life Technologies). 1 μg of total community DNA extracted from each of the three GM_SE samples were used for deep shotgun sequencing. While the 2010 and 2011 samples were sequenced by Ion Torrent Personal Genome Machine (Ion PGM) (Life Technologies, USA) using 200 bp read chemistry on Ion 318 Chips, for the 2012 sample the Ion Proton platform with 200 bp read chemistry on PI V2 Chip was used. As such, the 2010 and 2011 GM_SE samples were differentially barcoded and sequenced in tandem using three Ion 318 chips. The 2012 GM_SE sample, in its turn, was multiplexed with five other unrelated samples on a single PI V2 Chip. Quality of the relevant metagenomic DNA sample was checked by electrophoresis and determined to be of high quality with no visible degradation. Quantity was determined using Qubit dsDNA HS Assay Kit (Life Technologies).

The libraries for the **Ion Torrent** platform were constructed using the Ion Plus Fragment Library Kit (Life Technologies) and the experimental procedures followed the manufacturer’s Ion Plus gDNA and Amplicon Library Preparation User Guide. The PGM library was generated using 1 µg of genomic DNA which was fragmented to approximately 200 base pairs by the Covaris S2 system (Covaris, Inc., Woburn, MA, USA) and purified with 1.8x Agencourt Ampure XP Beads (Beckman Coulter). Fragmentation was followed by end-repair, blunt-end ligation of the Ion Xpress Barcode and Ion P1 adaptors as well as nick translation.

Post-ligation size selected using E-Gel Size- Select 2% Agarose gels (Life Technologies) with the target size of 330 bp. Final PCR was performed again using platinum PCR SuperMix High Fidelity and Library Amplification Primer Mix (custom product by Invitrogen), for 5 cycles of amplification. The ION Torrent PGM library was followed by AMPure XP bead purification (1.2x). An Agilent 2100 Bioanalyzer high-sensitivity DNA kit (Agilent, Santa Clara, CA) was used to visualize the size range and determine the library concentration. Libraries were pooled in equimolar concentrations and used for template preparation.

Library templates were prepared for sequencing using the Life Technologies Ion Xpress and Ion OneTouch protocols and reagents. Briefly, library fragments were clonally amplified onto ion sphere particles (ISPs) through emulsion PCR and then enriched for template-positive ISPs. PGM emulsion PCR reactions utilized the Ion OneTouch 200 Template Kit v2 DL (Life Technologies). Following recovery, enrichment was completed by selectively binding the ISPs containing amplified library fragments to streptavidin coated magnetic beads, removing empty ISPs through washing steps, and denaturing the library strands to allow for collection of the template-positive ISPs. For all reactions, these steps were accomplished using the Life Technologies ES module of the Ion OneTouch. The complete ISPs sample (Chip 318) was prepared for sequencing using the protocol Ion PGM 200 Sequencing Kit (Life Technologies). Sequencing was performed with the Ion PGM 200 Sequencing Kit (Life Technologies) using the 500 flow (125 cycle) run format.

The libraries for the **Ion Proton** platforms were constructed using the Ion Plus Fragment Library Kit (Life Technologies) and the experimental procedures followed the manufacturer’s Ion Plus gDNA and Amplicon Library Preparation User Guide. The Proton library was generated using 1 µg of genomic DNA which was fragmented to approximately 200 base pairs by the Covaris S2 system (Covaris, Inc., Woburn, MA, USA) and purified with 1.8x Agencourt Ampure XP Beads (Beckman Coulter). Fragmentation was followed by end-repair, blunt-end ligation of the Ion Xpress Barcode and Ion P1 adaptors as well as nick translation.

Post-ligation size selected using E-Gel Size- Select 2% Agarose gels (Life Technologies) with the target size of 300 bp. Final PCR was performed again using platinum PCR SuperMix High Fidelity and Library Amplification Primer Mix (custom product by Invitrogen), for 5 cycles of amplification. The resulting library was purified using AMPure XP reagent (1.2x; Beckman Coulter) and the concentration determined with Qubit dsDNA HS Assay Kit (Life Technologies) and the respective size distribution with Agilent 2100 Bioanalyzer high-sensitivity DNA kit (Agilent, Santa Clara, CA). Libraries were pooled in equimolar concentrations and used for template preparation.

Library templates were prepared for sequencing using the Life Technologies OneTouch 2 protocols and reagents. Briefly, library fragments were clonally amplified onto ion sphere particles (ISPs) through emulsion PCR and then enriched for template-positive ISPs. Proton emulsion PCR reactions utilized the Ion PI Template OT2 200 Kit v3 (Life Technologies). Following recovery, enrichment was completed by selectively binding the ISPs containing amplified library fragments to streptavidin coated magnetic beads, removing empty ISPs through washing steps, and denaturing the library strands to allow for collection of the template-positive ISPs. For all reactions, these steps were accomplished using the Life Technologies ES module of the Ion OneTouch 2. The complete ISPs sample was loaded on Chip PI V2 using the protocol Ion PI Sequencing 200 kit v3 (Life Technologies). Sequencing was performed with the Ion PI 200 Sequencing Kit (Life Technologies) using the 500 flow (125 cycle) run format. For the single run, the Proton P1 Chip was first pre-rinsed and incubated with NaOH for 1 min before loading in order to minimize residual contaminants and decrease background signal.

***Estimation of Ecological diversity from the shotgun metagenomic data***

Ecological diversity of the individual GM_SEs were calculated by rendering the phylum-level distribution of their shotgun metagenomic reads to standard mathematical indices that quantify community composition by accounting for the number of types (here ‘phyla’) present plus the relative abundance of the different types. Thus for the current calculations proportion of shotgun metagenomic reads affiliated to a particular phylum was taken as a measure of the relative abundance of that phylum in the given sample. As such, ecological diversity of every GM_SE edition was assessed using Simpson Dominance and, Shannon–Wiener Diversity and Evenness indices (Magurran, 2003).

In order to quantify the extent to which the various bacterial phyla dominated a given GM_SE edition we determined its Simpson index of dominance (*D*) following the equation 1. Here, n_i_ denoted the number of reads belonging to the i^th^ phylum (and S denoted the total number of phyla identified in the community sample in question); n was the total number of classifiable reads in the metagenome in question; and the (n_i_ / n) ratio, i.e. the proportion of representation of the i^th^ phylum in the entire community, was denoted as p_i_.

The Shannon diversity index (*H*) was measured by first calculating the p_i_ [or (n_i_ / n)] values as above, following which all p_i_ values were multiplied by their own natural logarithms (Ln p_i_). Finally, the resulting products were summed across phyla and multiplied by -1, as shown in equation 2.

For calculating the evenness of the community structure Shannon equitability index (*E_H_*) was calculated for a given GM_SE edition by dividing its *H* value by *H*_max_. *H*_max_ is known to be equal to Ln S, where S denoted the total number of phyla identified in the community in question. Equitability assumed a value between 0 and 1, with 1 representing complete evenness.

**
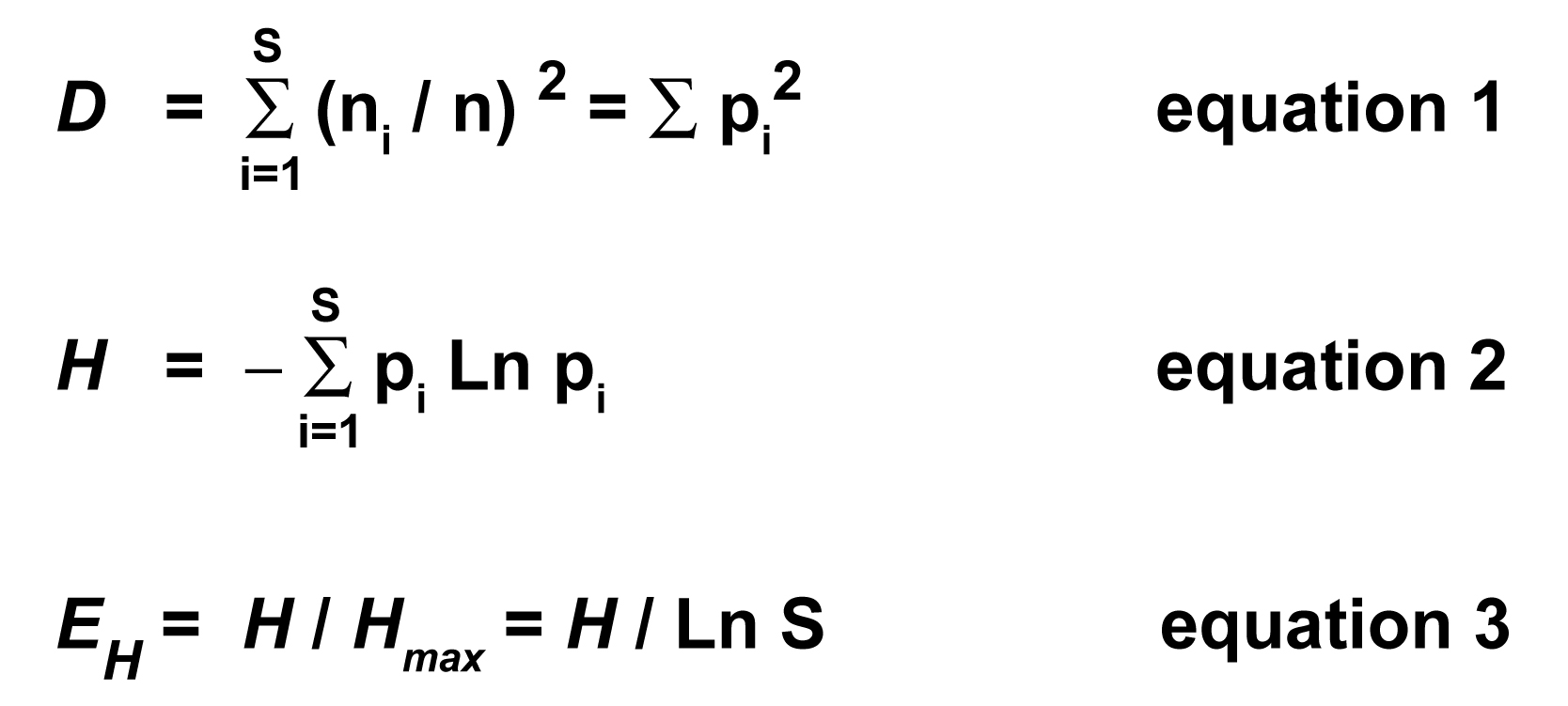
**

***Amplification of 16S rRNA gene fragments and sequencing by Ion PGM***

Amplification of 16S rRNA gene fragments and sequencing by Ion PGM was carried out using the Fusion Primer protocol. V3 regions of all potential bacterial or archaeal 16S rRNA genes present in the studied sample were amplified by polymerase chain reaction (PCR) using domain-specific oligonucleotide primers. In order to enable tandem sequencing of multiple samples on a single chip each sample DNA was subjected to PCR using a 16S forward primer prefixed with an Ion Torrent adapter and a unique sample-specific barcode or multiplex identifier in the following order in the 5’ to 3’ direction: (i) a 26-mer A-linker followed by a 4-mer A-linker key (bases represented in bold fonts), common for all sample primers, (ii) a 10-mer barcode unique to each sample primer followed by a common 3-mer barcode adaptor (all marked as stars), and finally (iii) the relevant domain-specific universal forward primer in its 5’ to 3’ direction (underlined bases). Reverse primers, in their turn, had (i) a common trP1 adapter (bases represented in italics), followed by (ii) the relevant domain-specific universal reverse primer in its 5’ to 3’ direction (underlined bases).

As such, V3 portions of all bacterial 16S rRNA genes present in a sample DNA were amplified using the forward primer 5’ – **CCA TCT CAT CCC TGC GTG TCT CCG ACT CAG** ***** *** *** *** ***CC TAC GGG AGG CAG CAG – 3’ (where the underlined portion represents the universal primer 341f) and the reverse primer 5’-*CCT CTC TAT GGG CAG TCG GTG AT*A TTA CCG CGG CTG CTG G - 3’ (where the underlined portion represents the universal primer 515r). For the amplification of archaeal 16S rRNA genes, we used the forward primer 5’ - **CCA TCT CAT CCC TGC GTG TCT CCG ACT CAG *** *** *** ***AA TTG GAN TCA ACG CCG G – 3’ (where the underlined portion represents the universal primer 344f) and the reverse primer 5’ - *CCT CTC TAT GGG CAG TCG GTG A*TC GRC GGC CAT GCA CCW C – 3’ (where the underlined portion represents the universal primer 522r).

Each 50 µl PCR reaction contained 10 µl template (corresponding to ~100 ng metagenomic DNA), 5 µl 10X KOD DNA polymerase buffer, 5 µl dNTP (0.25 mM each), 2 µl MgCl_2_ (25 mM), 1.5 µl (3%) DMSO, 3 µl each of the forward and reverse primer (0.3 µM each), 19.5 µl dH_2_O and 1 µl KOD hot start polymerase enzyme (Novagen, USA). PCR products were amplified for30 cycles as follows: 94 ^O^C for 15 s, 65 ^O^C for 30 s and 68 ^O^C for 60 s. After amplification, all PCR products were electrophoresed on 2.5% w/v agarose gel, purified by size selection, and adjusted to final concentrations of 10 ng μl^−1^ using molecular grade water. PCR products from all the six samples were pooled up at equal concentrations for subsequent Ion PGM sequencing.

Before Ion PGM sequencing, size distribution and DNA concentration in the pooled-up amplicon mixture was examined using a Bioanalyzer 2100 (Agilent Technologies, USA). The mixed sample was adjusted to a final concentration of 26 pM and attached to the surface of Ion Sphere Particles (ISPs) using an Ion Onetouch 200 Template kit (Life Technologies, USA) according to the manufacturer's instructions. Manual enrichment of the resulting ISPs resulted in >95% templated-ISPs, which were then sequenced on Ion 316 Chips using the Ion PGM (Ion Express Template 200 chemistry) for 500 flows that gives an expected average read length of >220 bp. Post sequencing, individual sequence reads were filtered by the PGM software to remove low quality and polyclonal sequences. Sequences matching the PGM 3′ adaptor were also automatically trimmed. All the data quality-filtered on the PGM were exported as fastq files for downstream applications.

**Supplementary Figures and Tables**

**
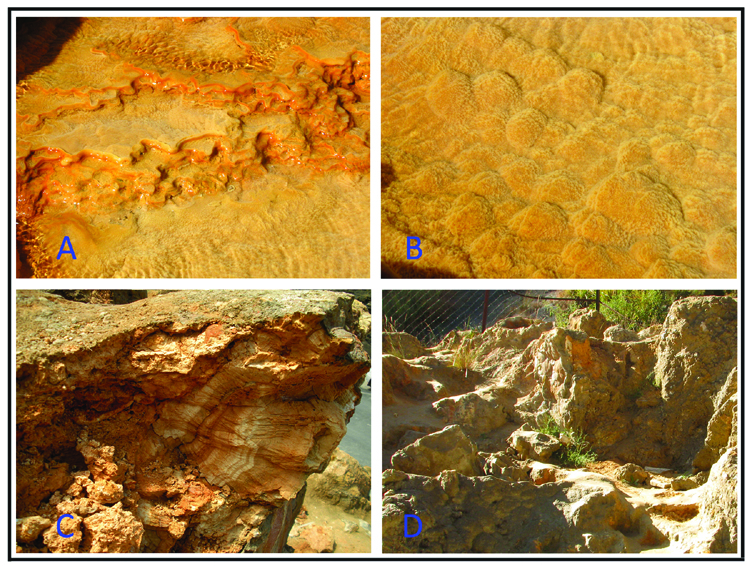
**

**Figure S1. Salient physical features of the Pilgrim Terrace sinter accretion observed in the three assessment years. A**, Active deposition of vertically-laminated red and creamy white sediments around the vent mouth on the flat Pilgrim Terrace top in 2010. **B**, Fluffy, fragile and bulbous yellow to red spherulites forming on the proximal hot water channels just below A. **C**, Sedimentary fabrics visible within the sinters layers exposed by fresh vertical cuts upon the travertine mound in 2011. **D**, Growth of angiospermous plants on the dwindling western slopes of the Pilgrim Terrace in 2012. All four photographs were taken by W. G.

**
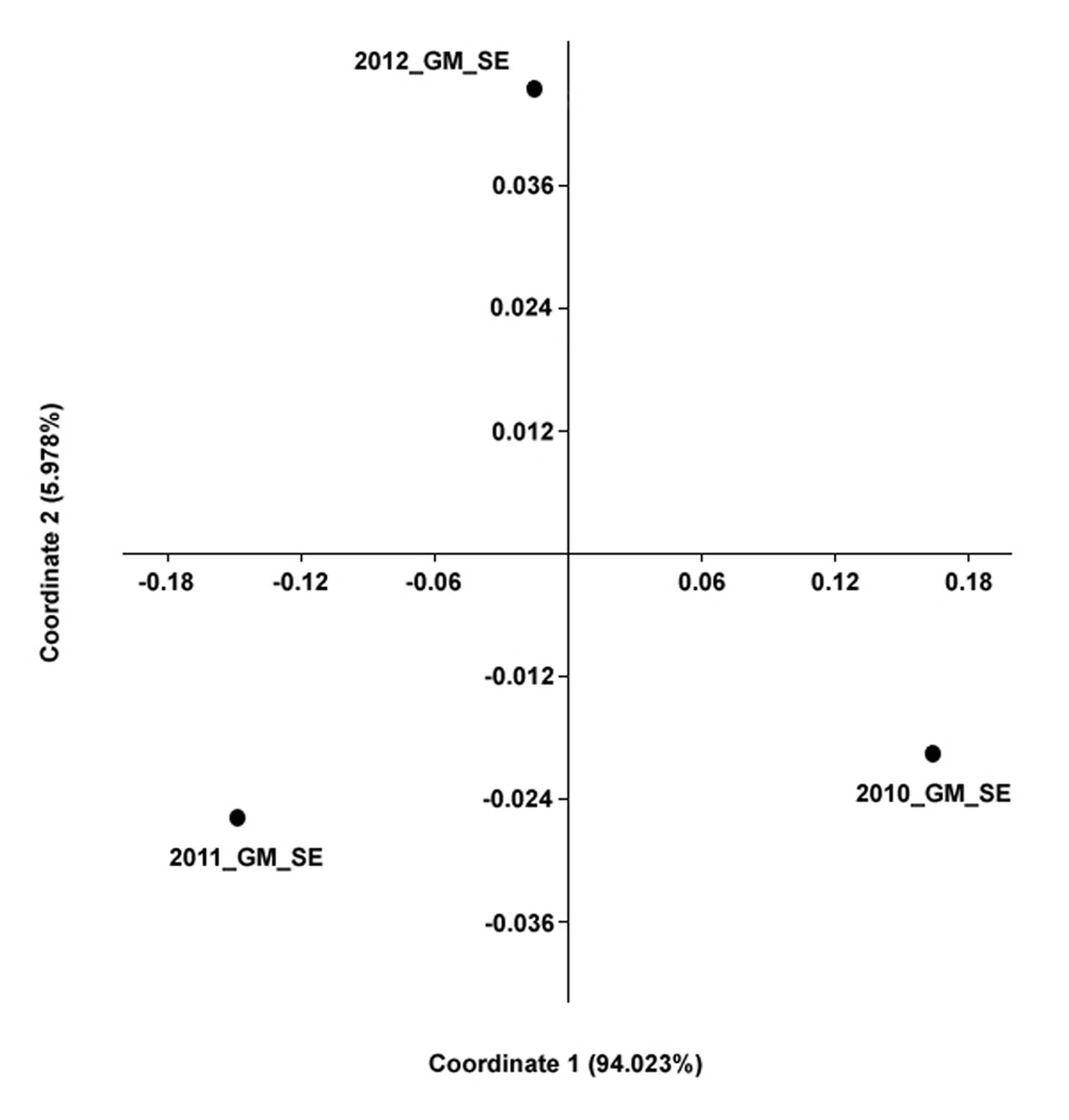
**

**Figure S2.** Principal Coordinate Analysis of the three GM_SE samples based on their physicochemical parameters and relative abundance of phyla. Bray-Curtis similarity matrix was used for this analysis. Each point corresponds to a microbial community, while percentages of variation explained by the plotted principal coordinates are indicated on the axes.

**
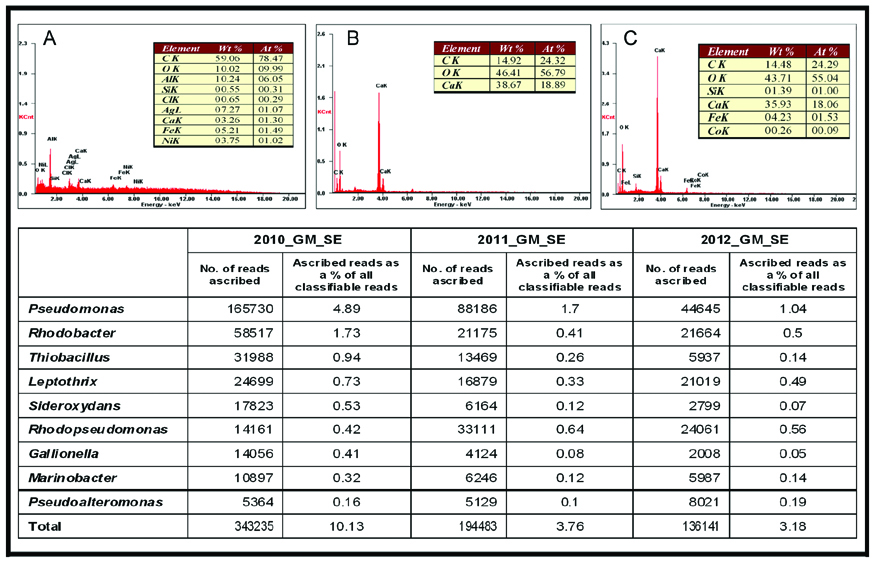
**

**Figure S3. Oscillation of the iron richness of the GM_SE substratum over the three assessment years and corresponding variations in the populations of major iron-oxidizing bacteria (IOB) in the three GM_SE editions.** Energy Dispersive X-ray Spectroscopy (EDS) was performed upon minute grains of the freshly precipitated sinter materials on which the three GM_SE samples grew. A through C respectively shows full-scan EDS spectra corresponding to 2010 through 2012 GM_SE, in that order. EDS was done in an EDAX system (attached to an FEI Quanta 200 scanning electron microscope) that detects the X-rays emitted from a sample during electron imaging. Quantitative analyses and atomic density ratios in the minerals encrusting the microfossils were worked out by the tools available in the GENESIS software controlling the EDAX. Metagenomic reads affiliated to IOB dominant in the three GM_SE editions have been tabulated below the EDS spectra.

**Table S1.** Summary statistics of all analyses done by MG-RAST upon the shotgun metagenomes of the three GM_SE editions.

|  | | **2010_GM_SE** | **2011_GM_SE** | **2012_GM_SE** |
| --- | --- | --- | --- | --- |
| **Primary data statistics** | Total no. of bases obtained | 902,498,992 | 1,618,827,271 | 1,340,692,381 |
|  | Mean read length | 152 ± 69 | 174 ± 75 | 159 ± 39 bp |
|  | Total no. of reads generated | 5,899,157 | 9,269,342 | 8,426,291 |
| **Read distribution over non-redundant protein database** | Total no. of reads annotated | 3,378,923 | 5,105,340 | 4,271,848 |
|  | Total no. of taxonomically-classifiable reads | 2,846,965 | 4,248,844 | 3,620,052 |
|  | Reads taken in by the 50 most-abundant genera | 2,290,880 | 2,386,918 | 2,089,938 |
|  | Total CDSs predicted | 767,650 | 4,393,706 | 4,716,978 |
|  | CDSs with assigned putative function | 499,482 | 2,180,350 | 1,768,123 |
|  | Total rRNAs predicted | 282,510 | 931,168 | 1,115,702 |
|  | rRNAs attributable to existing taxa | 1,358 | 5,632 | 9,386 |

**Table S2.** Phylum level distribution of bacterial metagenomic reads from the three GM_SE samples calculated using the “Organism Abundance” tool of MG-RAST. Reads were searched by blastx against the non-redundant (*nr*) protein database following the “Best Hit Classification” approach with minimum alignment length of 45 bp (15 amino acids) and minimum identity cutoff of 60%.

|  | **2010_GM_SE** | | **2011_GM_SE** | | **2012_GM_ SE** | |
| --- | --- | --- | --- | --- | --- | --- |
|  | **Read Count** | **Read Count as a % of total classifiable reads** | **Read Count** | **Read Count as a % of total classifiable reads** | **Read Count** | **Read Count as a % of total classifiable reads** |
| ***Proteobacteria*** | 2709993 | 95.39 | 1727313 | 41.28 | 2302034 | 64.63 |
| ***Actinobacteria*** | 76623 | 2.7 | 44331 | 1.06 | 60081 | 1.69 |
| ***Unclassified Bacteria*** | 11775 | 0.41 | 16449 | 0.39 | 11588 | 0.33 |
| ***Chloroflexi*** | 8798 | 0.31 | 270262 | 6.46 | 35218 | 0.99 |
| ***Bacteroidetes*** | 6310 | 0.22 | 470059 | 11.23 | 462031 | 12.97 |
| ***Cyanobacteria*** | 5290 | 0.19 | 712158 | 17.02 | 343173 | 9.63 |
| ***Firmicutes*** | 4206 | 0.15 | 125151 | 2.99 | 75417 | 2.12 |
| ***Nitrospirae*** | 3618 | 0.13 | 14931 | 0.36 | 8424 | 0.24 |
| ***Verrucomicrobia*** | 3535 | 0.12 | 70444 | 1.68 | 93061 | 2.61 |
| ***Chlorobi*** | 3214 | 0.11 | 23528 | 0.56 | 15580 | 0.44 |
| ***Deinococcus-Thermus*** | 3051 | 0.11 | 439904 | 10.51 | 35907 | 1.01 |
| ***Gemmatimonadetes*** | 1218 | 0.04 | 13545 | 0.32 | 7954 | 0.22 |
| ***Planctomycetes*** | 1029 | 0.04 | 93666 | 2.24 | 38302 | 1.08 |
| ***Spirochaetes*** | 507 | 0.02 | 12783 | 0.31 | 8714 | 0.24 |
| ***Deferribacteres*** | 482 | 0.02 | 2473 | 0.06 | 1782 | 0.05 |
| ***Acidobacteria*** | 432 | 0.02 | 109222 | 2.61 | 37895 | 1.06 |
| ***Poribacteria*** | 333 | 0.01 | 1769 | 0.04 | 922 | 0.03 |
| ***Chrysiogenetes*** | 197 | 0.01 | 1025 | 0.02 | 769 | 0.02 |
| ***Thermotogae*** | 151 | 0.01 | 6415 | 0.15 | 3107 | 0.09 |
| ***Synergistetes*** | 116 | 0.001 | 2992 | 0.07 | 1568 | 0.04 |
| ***Chlamydiae*** | 78 | 0.001 | 9106 | 0.22 | 8014 | 0.22 |
| ***Fusobacteria*** | 55 | 0.001 | 2264 | 0.05 | 1853 | 0.05 |
| ***Tenericutes*** | 44 | 0.001 | 88 | 0.001 | 785 | 0.02 |
| ***Aquificae*** | 39 | 0.001 | 9005 | 0.22 | 4695 | 0.13 |
| ***Elusimicrobia*** | 8 | 0.001 | 832 | 0.02 | 476 | 0.01 |
| ***Dictyoglomi*** | 0 | 0.000 | 1675 | 0.04 | 681 | 0.02 |
| ***Fibrobacteres*** | 0 | 0.000 | 534 | 0.01 | 417 | 0.01 |
| ***Lentisphaerae*** | 0 | 0.000 | 2264 | 0.05 | 1632 | 0.05 |

**Table S3A.** Calculation of ecological indices for 2010_GM_SE using phylum-level distribution of shotgun metagenomic reads.

| **Phyla** |  | **Dominance** | | **Shannon diversity index (H)** | |
| --- | --- | --- | --- | --- | --- |
|  | **Raw read count = n_i_** | **p_i_ = n_i_ / n** | **p_i_ ^2^** | **Ln p_i_** | **p_i_ (Ln p_i_)** |
| ***Proteobacteria*** | 2709993 | 0.95385 | 0.90983 | -0.047248853 | -0.04507 |
| ***Actinobacteria*** | 76623 | 0.02697 | 0.00073 | -3.613030142 | -0.09744 |
| **Unclassified Bacteria** | 11775 | 0.00414 | 0.00002 | -5.487059491 | -0.02272 |
| ***Chloroflexi*** | 8798 | 0.0031 | 0.00001 | -5.776353167 | -0.01791 |
| ***Bacteroidetes*** | 6310 | 0.00222 | 0 | -6.110248083 | -0.01356 |
| ***Cyanobacteria*** | 5290 | 0.00186 | 0 | -6.287178791 | -0.01169 |
| ***Firmicutes*** | 4206 | 0.00148 | 0 | -6.515713191 | -0.00964 |
| ***Nitrospirae*** | 3618 | 0.00127 | 0 | -6.668738379 | -0.00847 |
| ***Verrucomicrobia*** | 3535 | 0.00124 | 0 | -6.692643899 | -0.0083 |
| ***Chlorobi*** | 3214 | 0.00113 | 0 | -6.785537646 | -0.00767 |
| ***Deinococcus-Thermus*** | 3051 | 0.00107 | 0 | -6.840096631 | -0.00732 |
| ***Gemmatimonadetes*** | 1218 | 0.00043 | 0 | -7.751725349 | -0.00333 |
| ***Planctomycetes*** | 1029 | 0.00036 | 0 | -7.929406527 | -0.00285 |
| ***Spirochaetes*** | 507 | 0.00018 | 0 | -8.622553707 | -0.00155 |
| ***Deferribacteres*** | 482 | 0.00017 | 0 | -8.679712121 | -0.00148 |
| ***Acidobacteria*** | 432 | 0.00015 | 0 | -8.804875264 | -0.00132 |
| ***Poribacteria*** | 333 | 0.00012 | 0 | -9.028018815 | -0.00108 |
| ***Chrysiogenetes*** | 197 | 0.00007 | 0 | -9.567015316 | -0.00067 |
| ***Thermotogae*** | 151 | 0.00005 | 0 | -9.903487553 | -0.0005 |
| ***Synergistetes*** | 116 | 0.00004 | 0 | -10.1266311 | -0.00041 |
| ***Chlamydiae*** | 78 | 0.00003 | 0 | -10.41431318 | -0.00031 |
| ***Fusobacteria*** | 55 | 0.00002 | 0 | -10.81977828 | -0.00022 |
| ***Tenericutes*** | 44 | 0.00002 | 0 | -10.81977828 | -0.00022 |
| ***Aquificae*** | 39 | 0.00001 | 0 | -11.51292546 | -0.00012 |
| ***Elusimicrobia*** | 8 | 0 | 0 | undefined | Undefined |
| **N** | **2841102** |  |  |  |  |
| ***D* = Σ p_i_ ^2^** |  |  | **0.91059** |  |  |
| **Σ p_i_ (Ln p_i_)** |  |  |  |  | **-0.26385** |
| ***H* = – Σ p_i_ (Ln p_i_)** |  |  |  |  | **0.26385** |
| ***E_H_*= *H* / *H_max_*** |  |  |  |  | **0.08197** |

Shannon's equitability (*E_H_*) was obtained by dividing *H* by *H_max_*. *H_max_* is known to be Ln S, where S = total number of phyla in the community. Thus, here *H_max_* = Ln 25 = 3.218875825.

**Table S3B.** Calculation of ecological indices for 2011_GM_SE using phylum-level distribution of shotgun metagenomic reads.

| **Phyla** |  | **Dominance (*D*)** | | **Shannon diversity index (*H*)** | |
| --- | --- | --- | --- | --- | --- |
|  | **Raw read count = n_i_** | **p_i_ = n_i_ / n** | **p_i_ ^2^** | **Ln p_i_** | **p_i_ (Ln p_i_)** |
| ***Proteobacteria*** | 1727313 | 0.41282 | 0.17042 | -0.88474 | -0.36524 |
| ***Actinobacteria*** | 44331 | 0.01059 | 0.00011 | -4.54785 | -0.04816 |
| ***Unclassified Bacteria*** | 16449 | 0.00393 | 0.00002 | -5.53912 | -0.02177 |
| ***Chloroflexi*** | 270262 | 0.06459 | 0.00417 | -2.7397 | -0.17696 |
| ***Bacteroidetes*** | 470059 | 0.11234 | 0.01262 | -2.18623 | -0.2456 |
| ***Cyanobacteria*** | 712158 | 0.1702 | 0.02897 | -1.77078 | -0.30139 |
| ***Firmicutes*** | 125151 | 0.02991 | 0.00089 | -3.50956 | -0.10497 |
| ***Nitrospirae*** | 14931 | 0.00357 | 0.00001 | -5.63519 | -0.02012 |
| ***Verrucomicrobia*** | 70444 | 0.01684 | 0.00028 | -4.084 | -0.06877 |
| ***Chlorobi*** | 23528 | 0.00562 | 0.00003 | -5.18142 | -0.02912 |
| ***Deinococcus-Thermus*** | 439904 | 0.10513 | 0.01105 | -2.25256 | -0.23681 |
| ***Gemmatimonadetes*** | 13545 | 0.00324 | 0.00001 | -5.73218 | -0.01857 |
| ***Planctomycetes*** | 93666 | 0.02239 | 0.0005 | -3.79914 | -0.08506 |
| ***Spirochaetes*** | 12783 | 0.00306 | 0.00001 | -5.78934 | -0.01772 |
| ***Deferribacteres*** | 2473 | 0.00059 | 0 | -7.43539 | -0.00439 |
| ***Acidobacteria*** | 109222 | 0.0261 | 0.00068 | -3.64582 | -0.09516 |
| ***Poribacteria*** | 1769 | 0.00042 | 0 | -7.77526 | -0.00327 |
| ***Chrysiogenetes*** | 1025 | 0.00024 | 0 | -8.33487 | -0.002 |
| ***Thermotogae*** | 6415 | 0.00153 | 0 | -6.48249 | -0.00992 |
| ***Synergistetes*** | 2992 | 0.00072 | 0 | -7.23626 | -0.00521 |
| ***Chlamydiae*** | 9106 | 0.00218 | 0 | -6.12843 | -0.01336 |
| ***Fusobacteria*** | 2264 | 0.00054 | 0 | -7.52394 | -0.00406 |
| ***Tenericutes*** | 88 | 0.00002 | 0 | -10.8198 | -0.00022 |
| ***Aquificae*** | 9005 | 0.00215 | 0 | -6.14229 | -0.01321 |
| ***Elusimicrobia*** | 832 | 0.0002 | 0 | -8.51719 | -0.0017 |
| ***Dictyoglomi*** | 1675 | 0.0004 | 0 | -7.82405 | -0.00313 |
| ***Fibrobacteres*** | 534 | 0.00013 | 0 | -8.94798 | -0.00116 |
| ***Lentisphaerae*** | 2264 | 0.00054 | 0 | -7.52394 | -0.00406 |
| **N** | **4184188** |  |  |  |  |
| ***D* = Σ p_i_ ^2^** |  |  | **0.22977** |  |  |
| **Σ p_i_ (Ln p_i_)** |  |  |  |  | **-1.90111** |
| ***H* = – Σ p_i_ (Ln p_i_)** |  |  |  |  | **1.90111** |
| ***E_H_*= *H* / *H_max_*** |  |  |  |  | **0.57053** |

Shannon's equitability (*E_H_*) was obtained by dividing *H* by *H_max_*. *H_max_* is known to be Ln S, where S = total number of phyla in the community. Thus, here *H_max_* = Ln 28 = 3.33220451.

**Table S3C.** Calculation of ecological indices for 2012_GM_SE using phylum-level distribution of shotgun metagenomic reads.

|  |  | **Dominance (*D*)** | | **Shannon diversity index (*H*)** | |
| --- | --- | --- | --- | --- | --- |
|  | **Raw read count** | **n_i_ / n** | **(n_i_ / n)^2^** | **log (n_i_ / n)** | **(n_i_ / n) [log (n_i_ / n)]** |
| *Proteobacteria* | 2302034 | 0.64626 | 0.41765 | -0.43655 | -0.28213 |
| *Actinobacteria* | 60081 | 0.01687 | 0.00028 | -4.08222 | -0.06887 |
| *Unclassified Bacteria* | 11588 | 0.00325 | 0.00001 | -5.7291 | -0.01862 |
| *Chloroflexi* | 35218 | 0.00989 | 0.0001 | -4.61623 | -0.04565 |
| *Bacteroidetes* | 462031 | 0.12971 | 0.01682 | -2.04245 | -0.26493 |
| *Cyanobacteria* | 343173 | 0.09634 | 0.00928 | -2.33987 | -0.22542 |
| *Firmicutes* | 75417 | 0.02117 | 0.00045 | -3.85517 | -0.08161 |
| *Nitrospirae* | 8424 | 0.00236 | 0.00001 | -6.04909 | -0.01428 |
| *Verrucomicrobia* | 93061 | 0.02613 | 0.00068 | -3.64467 | -0.09524 |
| *Chlorobi* | 15580 | 0.00437 | 0.00002 | -5.43299 | -0.02374 |
| *Deinococcus-Thermus* | 35907 | 0.01008 | 0.0001 | -4.5972 | -0.04634 |
| *Gemmatimonadetes* | 7954 | 0.00223 | 0 | -6.10575 | -0.01362 |
| *Planctomycetes* | 38302 | 0.01075 | 0.00012 | -4.53285 | -0.04873 |
| *Spirochaetes* | 8714 | 0.00245 | 0.00001 | -6.01167 | -0.01473 |
| *Deferribacteres* | 1782 | 0.0005 | 0 | -7.6009 | -0.0038 |
| *Acidobacteria* | 37895 | 0.01064 | 0.00011 | -4.54313 | -0.04834 |
| *Poribacteria* | 922 | 0.00026 | 0 | -8.25483 | -0.00215 |
| *Chrysiogenetes* | 769 | 0.00022 | 0 | -8.42188 | -0.00185 |
| *Thermotogae* | 3107 | 0.00087 | 0 | -7.04702 | -0.00613 |
| *Synergistetes* | 1568 | 0.00044 | 0 | -7.72874 | -0.0034 |
| *Chlamydiae* | 8014 | 0.00225 | 0.00001 | -6.09683 | -0.01372 |
| *Fusobacteria* | 1853 | 0.00052 | 0 | -7.56168 | -0.00393 |
| *Tenericutes* | 785 | 0.00022 | 0 | -8.42188 | -0.00185 |
| *Aquificae* | 4695 | 0.00132 | 0 | -6.63012 | -0.00875 |
| *Elusimicrobia* | 476 | 0.00013 | 0 | -8.94798 | -0.00116 |
| *Dictyoglomi* | 681 | 0.00019 | 0 | -8.56849 | -0.00163 |
| *Fibrobacteres* | 417 | 0.00012 | 0 | -9.02802 | -0.00108 |
| *Lentisphaerae* | 1632 | 0.00046 | 0 | -7.68428 | -0.00353 |
| **N** | **3562080** |  |  |  |  |
| ***D* = Σ p_i_ ^2^** |  |  | **0.44565** |  |  |
| **Σ p_i_ (Ln p_i_)** |  |  |  |  | **-1.34523** |
| ***H* = – Σ p_i_ (Ln p_i_)** |  |  |  |  | **1.34523** |
| ***E_H_*= *H* / *H_max_*** |  |  |  |  | **0.40371** |

Shannon's equitability (*E_H_*) was obtained by dividing *H* by *H_max_*. *H_max_* is known to be Ln S, where S = total number of phyla in the community. Thus, here *H_max_* = Ln 28 = 3.33220451.

**Table S4.** Pairwise Bray-Curtis similarity (shaded green) and Euclidean distance (shaded red) values obtained by correlating the three GM_SE editions using phylum-level metagenomic data.

|  | **2010_GM_SE** | **2011_GM_SE** | **2012_GM_SE** |
| --- | --- | --- | --- |
| **2010_GM_SE** | - | 130.95 | 95.67 |
| **2011_GM_SE** | 0.69 | - | 41.72 |
| **2012_GM_SE** | 0.81 | 0.85 | - |

**Table S5.** List of the homologs of methanogenesis-associated genes that were identified in the shotgun metagenomes of the three GM_SE editions.

| **2010_GM_SE (6)** | **2011_GM_SE (10)** | **2012_GM_SE (16)** |
| --- | --- | --- |
| Fmd = Formylmethanofuran dehydrogenase  Subunit A (EC 1.2.99.5) | Fmd = Formylmethanofuran dehydrogenase  subunit A (EC 1.2.99.5) | Fmd = Formylmethanofuran dehydrogenase  subunit A (EC 1.2.99.5) |
| **-** | **-** | Formylmethanofuran dehydrogenase subunit B (EC 1.2.99.5) |
| Formylmethanofuran dehydrogenase  Subunit C (EC 1.2.99.5) | **-** | **-** |
| Formylmethanofuran—tetrahydromethanopterin  N-formyltransferase (EC 2.3.1.101) | Formylmethanofuran--tetrahydromethanopterin N-formyltransferase (EC 2.3.1.101) | Formylmethanofuran--tetrahydromethanopterin N-formyltransferase (EC 2.3.1.101) |
| N(5),N(10)-methenyltetrahydromethanopterin  cyclohydrolase (EC 3.5.4.27) | N(5),N(10)-methenyltetrahydromethanopterin cyclohydrolase (EC 3.5.4.27) | N(5),N(10)-methenyltetrahydromethanopterin cyclohydrolase (EC 3.5.4.27) |
| **-** | F_420_-dependent N(5),N(10)-methylenetetrahydromethanopterin reductase (EC 1.5.99.11) | F_420_-dependent N(5),N(10)-methylenetetrahydromethanopterin reductase (EC 1.5.99.11) |
| **-** | **-** | Coenzyme F(420):H(2) dehydrogenase (methanophenazine) subunits FpoJ |
| **-** | **-** | Coenzyme F(420):H(2) dehydrogenase (methanophenazine) subunits FpoK |
| **-** | Coenzyme F(420):H(2) dehydrogenase (methanophenazine) subunit FpoL | Coenzyme F(420):H(2) dehydrogenase (methanophenazine) subunits FpoL |
| **-** | **-** | Coenzyme F(420):H(2) dehydrogenase (methanophenazine) subunits FpoN |
| **-** | CoB--CoM heterodisulfide reductase subunit A (EC 1.8.98.1) | CoB--CoM heterodisulfide reductase subunit A (EC 1.8.98.1) |
| **-** | CoB--CoM heterodisulfide reductase subunit B (EC 1.8.98.1) | CoB--CoM heterodisulfide reductase subunit B (EC 1.8.98.1) |
| **-** | CoB--CoM heterodisulfide reductase subunit C (EC 1.8.98.1) | CoB--CoM heterodisulfide reductase subunit C (EC 1.8.98.1) |
| **-** | **-** | CoB--CoM-reducing hydrogenase (Cys) alpha subunit |
| Monomethylamine permease  (Methanogenesis from methylated compounds) | Monomethylamine permease  (Methanogenesis from methylated compounds) | Monomethylamine permease  (Methanogenesis from methylated compounds) |
| Trimethylamine:corrinoid methyltransferase  (Methanogenesis from methylated compounds) | Trimethylamine:corrinoid methyltransferase  (Methanogenesis from methylated compounds) | Trimethylamine:corrinoid methyltransferase  (Methanogenesis from methylated compounds) |
| **-** | **-** | Methanol:corrinoid methyltransferase (Methanogenesis from methylated compounds) |

**Table S6.** Methylotrophic and methanotrophic genera that occurred in the list of the top-50 genera of the three GM_SE editions. Information about the genera in question has been compiled from Bowman (2006) ^8^.

|  | **2010_GM_SE** | **2011_GM_SE** | **2012_GM_SE** |
| --- | --- | --- | --- |
| ***Methylibium*** (A facultatively methylotrophic betaproteobacterium that uses methanol as a sole carbon source, and also grow heterotrophically in defined media containing ethanol, toluene, benzene, ethylbenzene and dihydroxybenzoates as sole carbon sources) | + | + | + |
| ***Methylobacillus*** (A 1-C utilizing methanotrophs belonging to the family *Methylophilaceae* of *Betaproteobacteria*) | + | + | + |
| ***Methylobacterium*** (A facultatively methylotrophic aphaproteobacterium belonging to the family *Methylobacteriaceae*. It can grow on 1-C compounds such as formate, formaldehyde & methanol as sole source of carbon and energy as well as on a wide range of multicarbon growth substrates. Most, but not all, strains can grow on nutrient agar and some can grow on methylated amines. Only one strain has been reported to utilize methane as sole carbon source.) | + | + | + |
| ***Methylotenera*** (A 1-C utilizing methanotrophs belonging to the family *Methylophilaceae* of *Betaproteobacteria*) | + | - | + |
| ***Methylovorus*** (A 1-C utilizing methanotrophs belonging to the family *Methylophilaceae* of *Betaproteobacteria*) | + | - | - |
| ***Methylococcus*** (Type I methanotroph belonging to *Methylococcaceae* of *Gammaproteobacteria*. They possess a highly specialized metabolism restricted to the utilization of methane & methanol & are a subset of the methylotrophs, bacteria and archaea able to utilize C1 compounds. These are by definition obligately methylotrophic and do not have the ability to grow on organic compounds possessing carbon-carbon bonds. Besides methane, the only other substrate generally utilized for growth is methanol; however, a few strains can utilize methylamine and a narrow selection of other C1 compounds. | + | - | - |

**Table S7.** Numerical details of OTU analysis of the three GM_SE and two GM_PC samples.

|  |  | **2010_GM_SE** | **2010_GM_PC** | **2011_GM_SE** | **2012_GM_SE** | **2012_GM_PC** |
| --- | --- | --- | --- | --- | --- | --- |
| **Raw data** | Total reads | 235,748 | 169,739 | 119,130 | 187,643 | 242,756 |
| **Preprocessing of reads** | Reads after filtering at Q20 & Length 100 | 174,755 | 140,593 | 91,176 | 133,536 | 161,093 |
|  | Reads after preproc analysis | 54,207 | 42,378 | 40,661 | 42,049 | 54,450 |
|  | Reads after Chimera analysis | 47,580 | 35,735 | 32,767 | 37,373 | 48,376 |
| **Denoising of reads** | Reads after Denoising stage | 7,252 | 6,658 | 7,611 | 5,964 | 10,778 |
| **OTU formation** | Total OTUs formed | 5,567 | 5,449 | 6,190 | 4,520 | 8,344 |
| **OTUs distribution** | Singletons | 4,089 | 4,379 | 4,942 | 3,300 | 6,321 |
|  | Doubletons | 1,293 | 942 | 1098 | 1021 | 1,663 |
|  | Tripletons | 168 | 119 | 130 | 175 | 316 |
|  | OTUs with 4 reads | 14 | 7 | 17 | 23 | 39 |
|  | OTUs with 5 reads | 1 | 2 | 3 | 1 | 4 |
|  | OTUs with more than 5 reads | 2 | 0 | 0 | 0 | 1 |
|  | OTUs count  (minus singletons) | 1478 | 1,070 | 1,248 | 1,220 | 2,023 |
| **Statistical estimations** | ACE estimation  (minus singletons) | 1478 | 1070 | 1248 | 1220 | 2023 |
|  | Shannon index  (minus singletons) | 6.489967 | 6.9622930 | 7.1142550 | 7.0888740 | 7.5925020 |
|  | Simpson index  (minus singletons) | 0.998444 | 0.9990371 | 0.9991705 | 0.9991472 | 0.9994831 |

**Table S8.** Number of OTUs identified at the phylum level in the three GM_SE editions.

|  | **2010_GM_SE** | **2010_GM_PC** | **2011_GM_SE** | **2012_GM_SE** | **2012_GM_PC** |
| --- | --- | --- | --- | --- | --- |
| ***Actinobacteria*** | 1 | 11 | 6 | 1 | 10 |
| ***Spirochaetes*** | 2 | 4 | 6 | 3 | 11 |
| ***Bacteroidetes*** | 207 | 85 | 206 | 85 | 254 |
| ***Proteobacteria*** | 768 | 281 | 492 | 716 | 806 |
| ***Cyanobacteria*** | 24 | 18 | 1 | 6 | 93 |
| ***Nitrospirae*** | 1 | 4 | 18 | 0 | 6 |
| ***Acidobacteria*** | 1 | 4 | 10 | 2 | 14 |
| ***Aquificae*** | 1 | 1 | 1 | 0 | 1 |
| ***Deinococcus-Thermus*** | 6 | 244 | 154 | 5 | 68 |
| ***Firmicutes*** | 0 | 54 | 3 | 5 | 19 |
| ***Chloroflexi*** | 0 | 37 | 7 | 1 | 19 |
| ***Armatimonadetes*** | 0 | 0 | 1 | 1 | 0 |
| ***Verrucomicrobia*** | 0 | 2 | 3 | 1 | 19 |
| ***Chlamydiae*** | 0 | 0 | 0 | 0 | 6 |
| **TM7** | 0 | 0 | 1 | 0 | 0 |
| **WS3** | 0 | 0 | 1 | 0 | 0 |
| ***Gemmatimonadetes*** | 0 | 0 | 1 | 0 | 2 |
| **unclassified_Bacteria** | 467 | 325 | 337 | 394 | 695 |
| **Total OTUs**  **minus singletons** | 1478 | 1070 | 1248 | 1220 | 2023 |

**Table S9.** Number of OTUs identified upto the class level in the three GM_SE editions.

|  | **2010_GM_OFC_SE** | **2011_GM_OFC_SE** | **2012_GM_OFC_SE** |
| --- | --- | --- | --- |
| ***Deinococci*** | 6 | 154 | 5 |
| ***Alphaproteobacteria*** | 219 | 148 | 186 |
| ***Gammaproteobacteria*** | 12 | 125 | 53 |
| ***Betaproteobacteria*** | 274 | 90 | 224 |
| ***Flavobacteria*** | 0 | 59 | 0 |
| ***Sphingobacteria*** | 80 | 54 | 46 |
| ***Nitrospirae*** | 1 | 18 | 0 |
| ***Spirochaetes*** | 2 | 6 | 3 |
| ***Actinobacteria*** | 1 | 6 | 1 |
| ***Chloroflexi*** | 0 | 5 | 1 |
| ***Acidobacteria_Gp3*** | 0 | 5 | 0 |
| ***Deltaproteobacteria*** | 0 | 4 | 2 |
| ***Bacilli*** | 0 | 3 | 4 |
| ***Acidobacteria_Gp6*** | 0 | 3 | 0 |
| ***Opitutae*** | 0 | 3 | 0 |
| ***Acidobacteria_Gp4*** | 1 | 2 | 2 |
| ***Cyanobacteria*** | 16 | 1 | 3 |
| ***Aquificae*** | 1 | 1 | 0 |
| ***Bacteroidetes*_incertae_sedis** | 0 | 1 | 0 |
| ***Gemmatimonadetes*** | 0 | 1 | 0 |
| ***Anaerolineae*** | 0 | 1 | 0 |
| ***Thermomicrobia*** | 0 | 1 | 0 |
| ***Chloroplast*** | 3 | 0 | 3 |
| ***Negativicutes*** | 0 | 0 | 1 |

**Table S10.** Number of OTUs identified upto the genus level in the three GM_SE editions

| **Genera identified** | **2010_GM_SE** | **2011_GM_SE** | **2012_GM_SE** |
| --- | --- | --- | --- |
| *Acidovorax* | **0** | **2** | **0** |
| *Acinetobacter* | **0** | **0** | **1** |
| *Advenella* | **0** | **1** | **0** |
| *Algoriphagus* | **0** | **1** | **2** |
| *Aquabacterium* | **2** | **1** | **1** |
| Armatimonadetes_gp5 | **0** | **1** | **1** |
| *Bdellovibrio* | **0** | **1** | **0** |
| *Bosea* | **0** | **1** | **1** |
| *Brevundimonas* | **1** | **7** | **1** |
| *Chitinimonas* | **0** | **1** | **0** |
| *Chloroflexus* | **0** | **5** | **1** |
| *Chryseobacterium* | **0** | **1** | **0** |
| *Curvibacter* | **0** | **1** | **0** |
| *Cytophaga* | **0** | **6** | **0** |
| *Elioraea* | **0** | **1** | **0** |
| *Ferruginibacter* | **0** | **1** | **0** |
| *Flavobacterium* | **0** | **2** | **0** |
| *Flexibacter* | **1** | **1** | **2** |
| *Fluviicola* | **0** | **1** | **0** |
| *Gemmatimonas* | **0** | **1** | **0** |
| *Gemmobacter* | **0** | **0** | **0** |
| Gp3 (*Acidobacteria*) | **0** | **5** | **0** |
| Gp4 (*Acidobacteria*) | **1** | **2** | **2** |
| Gp6 (*Acidobacteria*) | **0** | **3** | **0** |
| GpI (*Cyanobacteria*) | **1** | **1** | **0** |
| GpIV (*Cyanobacteria*) | **0** | **0** | **1** |
| *Gulbenkiania* | **0** | **0** | **1** |
| *Halomonas* | **0** | **1** | **0** |
| *Hydrocarboniphaga* | **0** | **1** | **0** |
| *Hydrogenobacter* | **1** | **1** | **0** |
| *Hydrogenophaga* | **0** | **0** | **5** |
| *Hyphomonas* | **2** | **0** | **1** |
| *Kineococcus* | **0** | **1** | **0** |
| *Lacibacter* | **0** | **1** | **0** |
| *Legionella* | **0** | **0** | **1** |
| *Leptonema* | **1** | **0** | **0** |
| *Limnobacter* | **47** | **1** | **34** |
| *Lysobacter* | **0** | **1** | **0** |
| *Marmoricola* | **0** | **1** | **0** |
| *Meiothermus* | **6** | **81** | **5** |
| *Methylophilus* | **0** | **2** | **0** |
| *Microvirga* | **1** | **0** | **0** |
| *Niabella* | **0** | **0** | **1** |
| *Nitrospira* | **1** | **18** | **0** |
| *Novispirillum* | **1** | **0** | **1** |
| *Ohtaekwangia* | **0** | **1** | **0** |
| *Opitutus* | **0** | **3** | **0** |
| *Paenibacillus* | **0** | **0** | **0** |
| *Parasegetibacter* | **0** | **1** | **0** |
| *Pelomonas* | **0** | **1** | **0** |
| *Phenylobacterium* | **1** | **1** | **1** |
| *Phyllobacterium* | **0** | **0** | **1** |
| *Pseudomonas* | **1** | **1** | **0** |
| *Pseudoxanthomonas* | **0** | **0** | **1** |
| *Ramlibacter* | **0** | **1** | **0** |
| *Rheinheimera* | **0** | **35** | **0** |
| *Roseateles* | **1** | **0** | **0** |
| *Runella* | **0** | **1** | **0** |
| *Sediminibacterium* | **0** | **0** | **0** |
| *Silanimonas* | **1** | **1** | **2** |
| *Streptococcus* | **0** | **0** | **2** |
| *Tepidimonas* | **1** | **1** | **6** |
| *Thermomicrobium* | **0** | **1** | **0** |
| *Thermomonas* | **0** | **1** | **0** |
| *Thermus* | **0** | **40** | **0** |
| TM7_genera_incertae_sedis | **0** | **1** | **0** |
| *Turneriella* | **1** | **6** | **3** |
| *Vampirovibrio* | **0** | **1** | **0** |
| *Veillonella* | **0** | **0** | **1** |
| WS3_genera_incertae_sedis | **0** | **1** | **0** |
| Total | **72** | **254** | **80** |

**Table S11.** Numerical details of OTU analysis of the 2011 and 2012 sediment samples.

|  |  | **2011_Sediment Sample** | **2012_Sediment Sample** |
| --- | --- | --- | --- |
| **Raw data** | Total reads | 74,517 | 157,884 |
| **Preprocessing of reads** | Reads after filtering at Q20 & Length 100 | 61,139 | 121,868 |
|  | Reads after preproc analysis | 15,972 | 22,505 |
|  | Reads after Chimera analysis | 13,874 | 21,099 |
| **Denoising reads** | Reads after Denoising stage | 1,955 | 2,996 |
| **OTU formation** | Total OTUs formed | 1,491 | 2,210 |
| **OTUs distribution** | Singletons | 1087 | 1,539 |
|  | Doubletons | 348 | 576 |
|  | Tripletons | 52 | 77 |
|  | OTUs with 4 reads | 4 | 16 |
|  | OTUs with 5 reads | 0 | 2 |
|  | OTUs with more than 5 reads | 0 | 0 |
|  | OTUs count (w/o singletons) | 404 | 671 |
| **Statistical estimations** | ACE estimation (w/o singletons) | 404 | 671 |
|  | Shannon index (w/o singletons) | 5.9873960 | 7.283633 |
|  | Simpson index (w/o singletons) | 0.9974463 | 0.9992998 |

**Table S12.** Number of OTUs identified upto the genus level in the 2011 and 2012 sediment samples.

| **Genera Identified** | **Number of OTUs in 2011_Sediment Sample** | **Number of OTUs in 2012_Sediment Sample** |
| --- | --- | --- |
| ***Planomicrobium*** | 23 | 9 |
| ***Paenisporosarcina*** | 7 | 9 |
| ***Planococcus*** | 4 | 0 |
| ***Propionibacterium*** | 1 | 1 |
| ***Dietzia*** | 1 | 1 |
| ***Riemerella*** | 1 | 0 |
| ***Limnobacter*** | 1 | 1 |
| ***Phyllobacterium*** | 1 | 0 |
| ***Sphingomonas*** | 1 | 1 |
| ***Escherichia/Shigella*** | 1 | 0 |
| ***Clostridium XI*** | 1 | 0 |
| ***Brevibacillus*** | 1 | 0 |
| ***Paenibacillus*** | 1 | 0 |
| ***Meiothermus*** | 0 | 5 |
| ***Brevundimonas*** | 0 | 2 |
| ***Pontibacter*** | 0 | 1 |
| ***Chryseobacterium*** | 0 | 1 |
| ***Acidovorax*** | 0 | 1 |
| ***Chloroflexus*** | 0 | 1 |

**References for Supplementary Materials**

**1. Dam, B., Mandal, S., Ghosh, W., Das Gupta, S.K. & Roy, P. The S4-intermediate pathway for the oxidation of thiosulfate by the chemolithoautotroph Tetrathiobacter kashmirensis and inhibition of tetrathionate oxidation by sulfite. *Res Microbiol* 158, 330-8 (2007).**

**2. Ghosh, W., Bagchi, A., Mandal, S., Dam, B. & Roy, P. Tetrathiobacter kashmirensis gen. nov., sp. nov., a novel mesophilic, neutrophilic, tetrathionate-oxidizing, facultatively chemolithotrophic betaproteobacterium isolated from soil from a temperate orchard in Jammu and Kashmir, India. *Int J Syst Evol Microbiol* 55, 1779-87 (2005).**

**3. Kelly, D.P. & Wood, A.P. Synthesis and Determination of Thiosulfate and Polythionates. *Methods in Enzymology* 243, 475-501 (1994).**

**4. Alam, M., Pyne, P., Mazumdar, A., Peketi, A. & Ghosh, W. Kinetic enrichment of 34S during proteobacterial thiosulfate oxidation and the conserved role of SoxB in S-S bond breaking. *Appl Environ Microbiol* 79, 4455-64 (2013).**

**5. Mazumdar, A. & Strauss, H. Sulfur and strontium isotopic compositions of carbonate and evaporite rocks from the late Neoproterozoic-early Cambrian Bilara Group (Nagaur-Ganganagar Basin, India): Constraints on intrabasinal correlation and global sulfur cycle. *Precambrian Research* 149, 217-230 (2006).**

**6. Herrera, L., Ruiz, P., Aguillon, J.C. & Fehrmann, A. A new spectrophotometric method for the determination of ferrous iron in the presence of ferric iron. *Journal of Chemical Technology & Biotechnology* 44, 171-181 (1989).**

**7. Magurran, A.E. *Measuring Biological Diversity*, (Blackwell Publishing Limited, Oxford, UK, 2003).**

**8. Bowman, J.P. The Methanotrophs - The Families Methylococcaceae and Methylocystaceae. in *The Prokaryotes*, Vol. 5 (eds. Dworkin, M., Falkow, S., Rosenberg, E., Schleifer, K.-H. & Stackebrandt, E.) 266-289 (Springer, New York, 2006).**
